# Cut from the same cloth: The convergent evolution of dwarf morphotypes of the *Carex flava* group (Cyperaceae) in Circum-Mediterranean mountains

**DOI:** 10.1371/journal.pone.0189769

**Published:** 2017-12-27

**Authors:** Pedro Jiménez-Mejías, Carmen Benítez-Benítez, Mario Fernández-Mazuecos, Santiago Martín-Bravo

**Affiliations:** 1 Department of Molecular Biology and Biochemical Engineering, Universidad Pablo de Olavide, Seville, Spain; 2 Department of Biodiversity and Conservation, Real Jardín Botánico (RJB-CSIC), Madrid, Spain; Indiana University Bloomington, UNITED STATES

## Abstract

Plants growing in high-mountain environments may share common morphological features through convergent evolution resulting from an adaptative response to similar ecological conditions. The *Carex flava* species complex (sect. *Ceratocystis*, Cyperaceae) includes four dwarf morphotypes from Circum-Mediterranean mountains whose taxonomic status has remained obscure due to their apparent morphological resemblance. In this study we investigate whether these dwarf mountain morphotypes result from convergent evolution or common ancestry, and whether there are ecological differences promoting differentiation between the dwarf morphotypes and their taxonomically related large, well-developed counterparts. We used phylogenetic analyses of nrDNA (ITS) and ptDNA (*rps*16 and 5’*trn*K) sequences, ancestral state reconstruction, multivariate analyses of macro- and micromorphological data, and species distribution modeling. Dwarf morphotype populations were found to belong to three different genetic lineages, and several morphotype shifts from well-developed to dwarf were suggested by ancestral state reconstructions. Distribution modeling supported differences in climatic niche at regional scale between the large forms, mainly from lowland, and the dwarf mountain morphotypes. Our results suggest that dwarf mountain morphotypes within this sedge group are small forms of different lineages that have recurrently adapted to mountain habitats through convergent evolution.

## Introduction

The adaptation of plant species to high-mountain environments frequently entails a series of convergent phenotypic traits that may be displayed by very different taxonomic groups. Geophytic or chasmophytic–frequently cushion-forming–growth forms, fleshy and/or tomentose leaves, CAM metabolism, and dwarfism are some of the characters that allow plants to survive in high-mountain ecosystems [1]. Morphological homoplasy induced by similar environmental conditions is frequently found in plants belonging to independently evolved lineages and at different taxonomic scales (Table 1), sometimes even confounding taxonomy (e.g. [2]). Some of these features are genetically determined, whereas others are the result of phenotypic plasticity, i.e. the interplay of genotype and environment (e.g. [3,4,5]; among others). In particular, dwarfism appears to be caused by both genetic and environmental causes [1]. Lower temperatures in mountains limit cell division and result in smaller plant sizes. Thus, alpine plants tend to have leaves that are, on average, one-tenth the size of those in conspecific lowland populations [6].

**Table 1:** Evolutionary studies showing cases of morphological homoplasy related to adaptation to mountain environments.

Reference	Convergent character	Taxa (Family)	Hypothesized adaptive function
[7]	Dwarf cushion-forming habit and white tomentose indumentum	*Veronica* spp. (Plantaginaceae)	Protection from low temperatures
[8]	Cushion-life form	Multiple appearances in Angiosperms	Resistance to low temperatures, freezing, and drought
[9]	Dwarf shrubby habit	*Alchemilla* spp. (Rosaceae)	Resistance to low temperatures and wind, microhabitat modification
[10]	Translucent bracts	*Rheum alexandrae* Batalin and *R*. *nobile* Hook.f. & Thomson (Polygonaceae)	Protection of the inflorescence, and pollen grains in particular, from low temperatures and ultraviolet light
[11]	Bright-colored bracteoles and thin stems	*Bupleurum commelynoideum* H.Boissieu s.l. (Apiaceae)	Protection of the flower buds from ultraviolet light and resistance to wind
[12,13]	Dwarf cushion-forming habit	*Androsace* spp. (Primulaceae)	Resistance to low temperatures.
[2]	Erect fronds with indeterminate growth and reduced pinnae	*Jamesonia* spp. (Pteridaceae)	Protection from soil-mediated freezing, maximization of photosynthetic activity at low temperatures
[14,15]	Elevated giant rosette growth	*Dendrosenecio* spp., *Espeletia* spp., *Argyroxiphium* spp. (Asteraceae), and *Lobelia* spp. (Campanulaceae)	Protection from soil-mediated freezing, insulation of flower buds, and water-storage
[16]	Downy buds	Multiple appearances in Angiosperms	Protection against low temperatures
[17]	Cushion-forming habit	*Arenaria* sect. *Plinthine* (Caryophyllaceae)	(none proposed)
This study	Dwarf habit	*Carex flava* L. and *C*. *lepidocarpa* Tausch. (Cyperaceae)	Resistance and protection from wind

**Fig 1:**
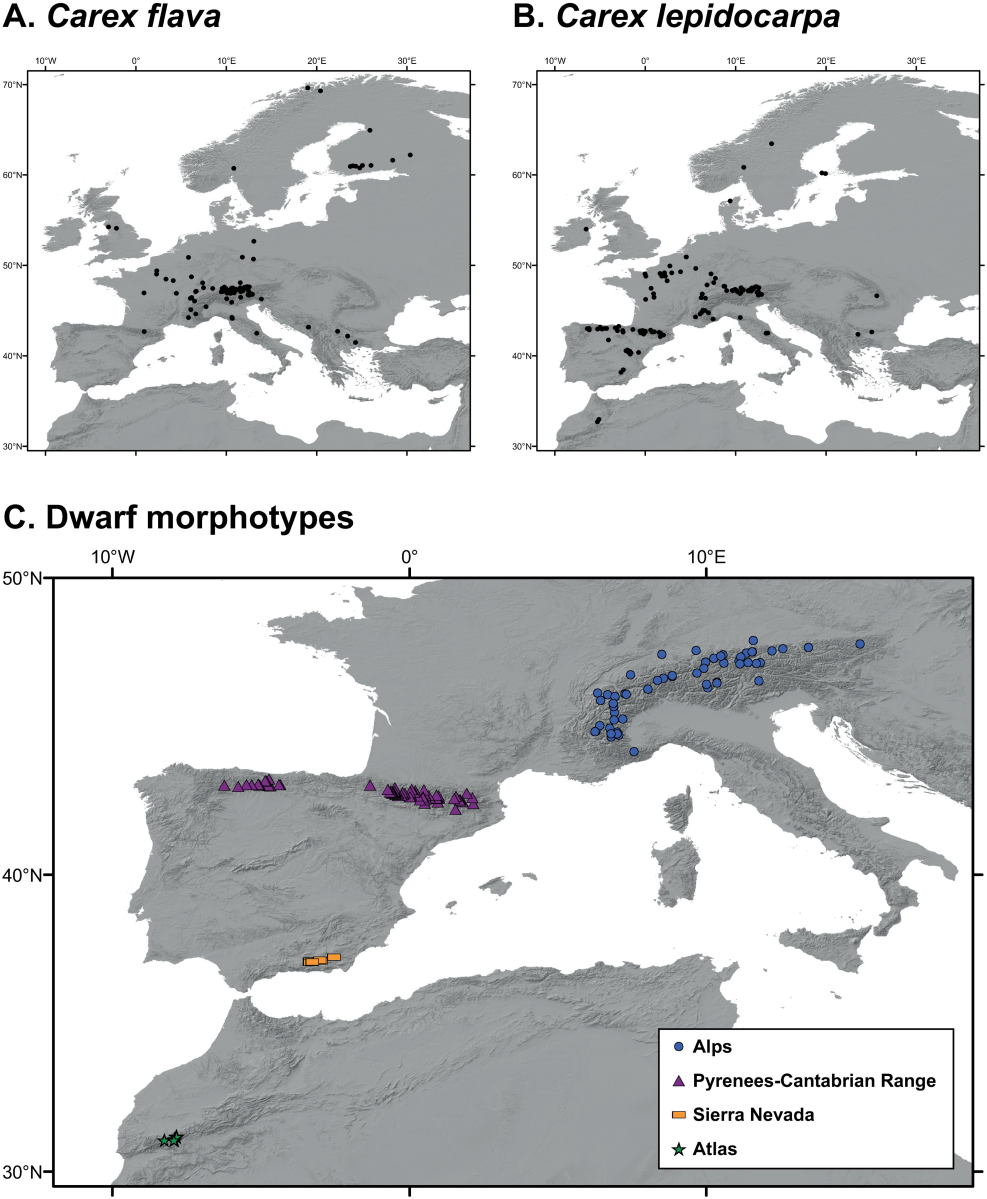
Distribution map of the localities included in the distribution modeling of well-developed species and dwarf morphotypes of the bent-beaked clade of the *Carex flava* group in the Mediterranean Basin. (A) *C*. *flava*, (B) *C*. *lepidocarpa*, (C) dwarf morphotypes: Alps (blue circles), Pyrenees-Cantabrian Range (N Iberia, purple triangles), Sierra Nevada (SE Iberia, yellow rectangles) and Atlas (NW Africa, green stars).

*Carex* sect. *Ceratocystis* Dumort. is a small group (5–19 species depending on the taxonomic treatment) of predominantly cespitose sedges mainly distributed in temperate Eurasia and North America [18]. Due to hybridization processes [19,20] and subtle morphological boundaries [21], this group displays a high degree of taxonomic complexity that has led many authors to generically refer to most of the taxa as the “*C*. *flava* group” ([22–28], among others). Recent works have established the existence of six well-defined species in sect. *Ceratocystis* in the Western Palaearctic: *C*. *castroviejoi* Luceño & Jim.-Mejías, *C*. *demissa* Hornem., *C*. *hostiana* DC., *C*. *flava* L., *C*. *lepidocarpa* Tausch. and *C*. *viridula* Michx. [18,21]. Apart from these well-defined species, there is a set of populations of dwarf morphotypes in some western and central Circum-Mediterranean mountains (High Atlas, Sierra Nevada, Pyrenees-Cantabrian Range, and Alps; Fig 1) whose taxonomic status remains disputed (Table 2). These are small-sized plants–usually no more than 10 cm high–that grow in mountain peat bogs and wet meadows. The differences between the dwarf and the well-developed morphotypes are known to be maintained under cultivation ([29]; Jiménez-Mejías pers. obs.). The strong morphological resemblance that the dwarf morphotypes share led Chater [30] to consider them all as a single species (*C*. *nevadensis* Boiss. & Reut.) in *Flora Europaea*. Morphological affinities of dwarf morphotypes with well-developed individuals of the well-defined species were investigated in a recent morphometric analysis [21]. Populations from the Sierra Nevada and High Atlas Mountains could not be distinguished from each other. The Pyrenean-Cantabrian populations were found to represent the smaller plants within the clinal variation of well-developed individuals of *C*. *lepidocarpa* and *C*. *flava*. Accordingly, the dwarf habit was identified as the main cause of the lack of discriminant characters. Similarly, Schmid [22] reported that dwarf plants from the Alps represent the smaller-sized portion of *C*. *flava* variation and considered the high altitude where these plants grow as the reason behind their different morphology. Molecular phylogenetic analyses [18] additionally showed that different mountain population sets have affinities with different species. This could suggest independent origins of the shared morphological features.

**Table 2:** Previous taxonomic treatments of the dwarf mountain morphotypes within the *Carex flava* group in Europe and Northwest Africa.

Geographical range	Previous treatments	Treatment according to the present study
Sierra Nevada (and Filabres Range; SE Spain)	*C*. *lepidocarpa* Tausch. [31]	*C*. *lepidocarpa* subsp. *nevadensis*
	*C*. *lepidocarpa* var. *nevadensis* (Boiss. & Reut.) Kük. [32,33]	
	*C*. *lepidocarpa* subsp. *nevadensis* (Boiss. & Reut.) Luceño [21]	
	*C*. *nevadensis* Boiss. & Reut. [28,30]	
Alps	*C*. *flava* L. [24]	*C*. *flava* and *C*. *lepidocarpa* (morphologically undistinguishable dwarf forms)
	*C*. *flava* var. *alpina* Kneuck. [22,29]	
	*C*. *nevadensis* [30]	
High Atlas	*C*. *flava* var. *nevadensis* [34]	*C*. *lepidocarpa* subsp. *ferraria* Jim.-Mejías & Martín-Bravo
	*C*. *nevadensis* [35]	
Pyrenees and Cantabrian Range	*C*. *demissa × C*. *lepidocarpa* [18,28]	*C*. *lepidocarpa* dwarf forms (with introgression from *C*. *demissa*)
	*C*. *flava* var. *alpina* [31,36]	
	*C*. *lepidocarpa* var. *nevadensis* [33]	
	*C*. *nevadensis* [37]	

Taxonomy has traditionally been based on macromorphological features, since most sensory information processed by the human brain is visual [38]. More rarely, taxonomy relies on characters perceived by other human senses, such as smell or flavour. Micromorphology and anatomy are additional sources of variation that may be used to distinguish macromorphologically cryptic taxa [39]. Micromorphological characters of achene epidermis have been studied for taxonomic purposes in *Carex*. They have clarified taxonomy in several species complexes (e.g., *Carex retrorsa* Schwein [40]; *Carex* sect. *Phacocystis* Dumort. [41]; *Carex* sect. *Phyllostachys* Tuck. [42]; *C*. *gynodynama* Olney and *C*. *mendocinensis* W.Boott [43]; former genus *Kobresia* Willd. [44]). Within *Carex* sect. *Ceratocystis*, achene epidermis has been also studied [23,45]. The detected variability has been reported to be linked to chromosome number variation, and thus to the taxonomic structure of the group.

The development of DNA-based barcoding methods has helped taxonomists detect and differentiate morphologically similar species, including cryptic taxa and species complexes (i.e. [38,46–50]. A phylogeny of sect. *Ceratocystis* based on nuclear ITS and plastid *rps*16 and 5’*trn*K sequences [18] showed that DNA sequences have high taxon specificity and discriminant power (e.g. 88.2% of the plastid haplotypes were taxon-specific), which is in accordance with findings in North American populations of taxa of the same section [51]. These results suggest that sequencing of specific regions may be useful to circumscribe taxa within *Carex* sect. *Ceratocystis*.

Species distribution modeling allows researchers to reconstruct the potential range of species/populations on the basis of climatic and other environmental variables [52,53]. This technique can be used to evaluate differences in ecological requirements between species and sets of populations. Currently, species distribution modeling and niche overlap analyses are being widely used in evolutionary studies to evaluate the role of climatic variables, together with geography, in the evolution of species and their ecological preferences [54,55].

In this paper, we use molecular, macromorphological and micromorphological data, as well as species distribution modeling, in the *C*. *flava* group to: 1) test the hypothesis of a lack of morphological differentiation between the sets of populations of dwarf morphotypes from different mountain ranges; 2) re-evaluate if the similar morphotypes are the result of convergence and how many times they have evolved; and 3) assess whether high mountain environments have induced the homoplasic morphological characteristics of these populations.

## Materials and methods

### Circumscription of study group

We considered four different population groups of dwarf mountain morphotypes within the *C*. *flava* group (Table 2; Fig 1): Alps, Pyrenees-Cantabrian Range, High Atlas (henceforth Atlas), and Sierra Nevada (see Introduction for further clarification). Samples from plants belonging to these groups were previously recovered in a clade also including *C*. *lepidocarpa* and *C*. *flava*, termed the bent-beaked clade [18]. In our phylogenetic study, we included samples of well-developed individuals ascribable to six well-defined taxa within sect. *Ceratocystis* (*C*. *demissa*, *C*. *flava*, *C*. *hostiana*, *C*. *lepidocarpa* subsp. *lepidocarpa*, *C*. *lepidocarpa* subsp. *jemtlandica*, and *C*. *viridula*).

We relied on materials already deposited in herbaria, as well as on field collections in Spanish territory. No permission was needed for field collections, as these did not include threatened species or new prospections in protected areas.

### Macromorphological study

We studied the morphological differentiation among the dwarf mountain morphotypes using classic multivariate techniques. The relationship between dwarf mountain morphotypes and well-developed individuals of the well-defined species were already studied in previous works [18,21,23,29]. Here, we intended to objectively assess the degree of morphological resemblance among the different population sets of the dwarf morphotypes.

One hundred specimens from herbaria (BM, JACA, LEB, M, MA, MSB, NEU, RNG) and field collections (deposited at UPOS) from the four groups of dwarf morphotypes were included in the macromorphological morphometric study: 17 specimens from Sierra Nevada, 21 from the Alps, six from the Atlas, and 56 from Pyrenees-Cantabrian Range (S1 Appendix). From a previous PCA exploration using 24 characters, we selected eight macromorphological quantitative characters (Table 3) as those with the highest correlation values with other characters. Measurements were made using an ocular micrometer, with an accuracy of up to 0.1 mm. All observations were performed using a stereoscopic binocular Nikon SMZ645 microscope. Glume and utricle color were scored as qualitative characters; these characters were not included in the multivariate analyses.

**Table 3:** Macro- and micromorphological characters studied for the dwarf populations of the *Carex flava* group. Loadings for the two principal components (PC-1 and PC-2) from MPCA (macromorphlogy) and mPCA (micromorphology) are provided; the highest loadings for each component are marked in bold.

**Macromorphology**				
**Character**	**Label**	**MPCA**		
		**PC-1**	**PC-2**	
Lowest bract length	LBL	0.764	0.321	
Lowest bract width	LBW	0.669	**0.503**	
Male spike width (mm)	MSW	0.152	**0.839**	
Male spike length / width ratio	MSR	0.675	-0.422	
Female glume mucro (mm)	FGM	-0.083	0.315	
Utricle length (mm)	UTL	**0.931**	-0.123	
Utricle width (mm)	UTW	0.691	-0.152	
Utricle beak length (mm)	UTB	**0.812**	-0.220	
**Micromorphology**				
**Character**	**Label**	**Calculation** (see Fig 2)	**mPCA**	
			**PC-1**	**PC-2**
Central body height	cbh	A/B	**-0.830**	0.443
Central body width	cbw	B/C	**0.879**	-0.061
Satellite bodies height	sbh	(((D+E)/2)-F)/B	-0.625	-0.161
Central body shape 1	cbs1	A/C	-0.111	**0.880**
Central body shape 2	cbs2	G/C	0.669	0.350
Inter-platforms gap width	igw	(H_1_+H_2_)/2	0.025	**0.453**

PCA was conducted to study the macromorphological variation (MPCA). Data were not transformed. Calculations were performed using a correlation matrix to minimize the effect of scale. Only principal components with eigenvalues greater than 1 were retained. Quartile distribution was calculated for each variable and morphotype to check the degree of overlapping. Characters were considered to be taxonomically useful when overlap was equal to or lower than a threshold of 25% [21,56]. All analyses were performed in PAWS Statistics 18.

### Micromorphological study

**Fig 2:**
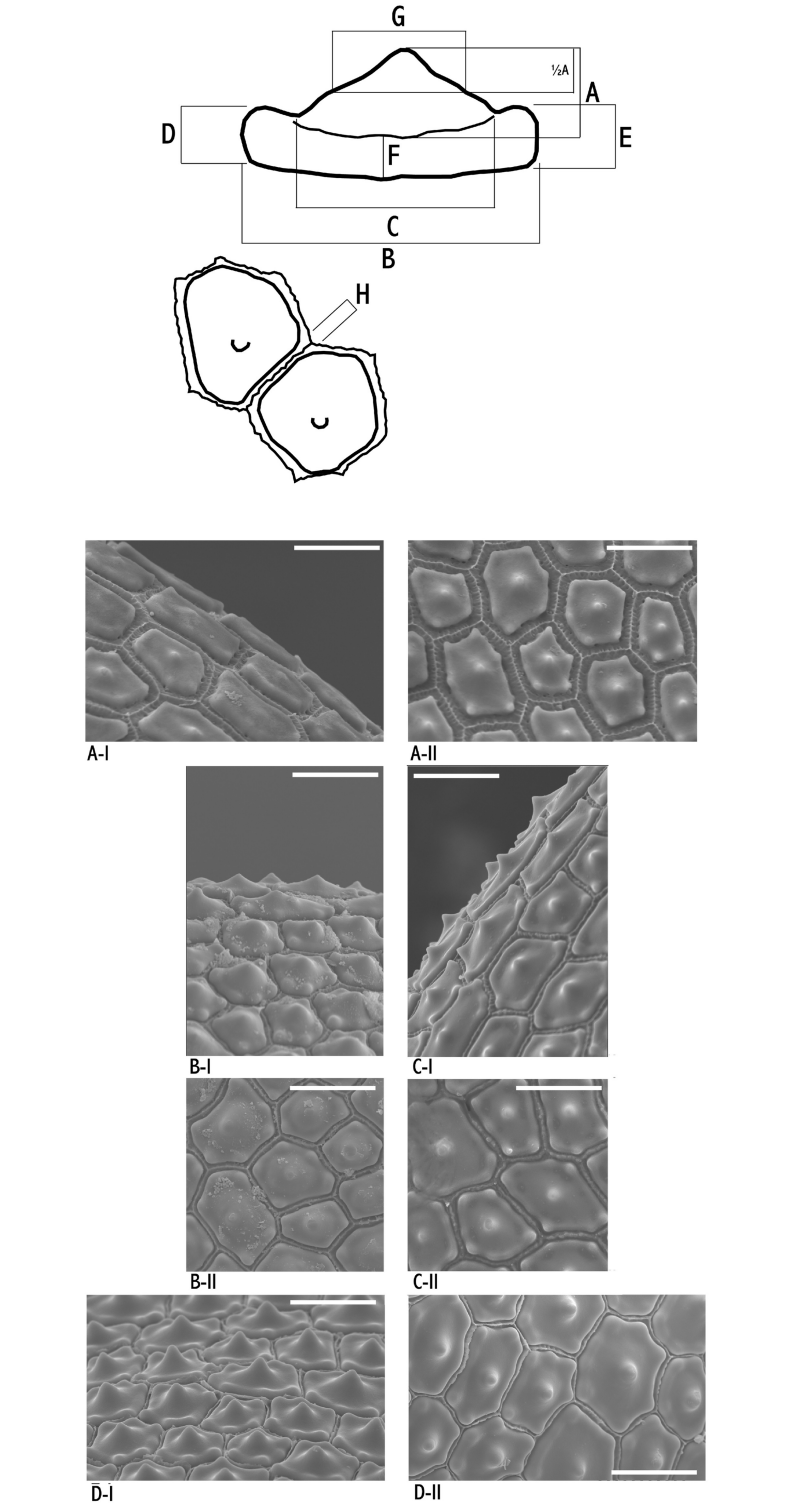
Above, schematic drawing of the measurements of the inner anticlinal wall and silica bodies of the achene epidermis cells in the *Carex flava* group (A-H) taken from SEM pictures. (A) Lateral view; (B) Overhead view. Below, SEM photographs (A, lateral shot; B, overhead perspective) of the wall of the achene of *C*. *flava* group dwarf morphotypes. Sample numbering according to S1 Appendix: (A) Alps morphotype (19); (B) Atlas morphotype (5); (C) Sierra Nevada morphotype (16); (D) Pyrenees-Cantabrian Range morphotype (12).

Silica bodies in achene epidermal cells were studied in search of additional features that may help distinguish the different sets of dwarf populations. Forty-three achenes from the four population groups of dwarf morphotypes were studied: ten from Sierra Nevada, 17 from the Alps, three from the Atlas and 13 from Pyrenees-Cantabrian Range (S1 Appendix). All studied specimens except three were also included in the macromorphological study. Although most achenes were taken from different vouchers, scarcity of ripe fruits forced us to include several achenes from the same voucher in a few cases. In order to visualize silica bodies, achenes were digested in a solution of acetic anhydride and sulfuric acid (9:1) for 24 h at room temperature, washed with distilled water, and then subjected to a 10 min ultrasonic bath in a Nahita 621/2 sonicator. Finally, achenes were placed in Petri dishes and air-dried at room temperature. Sonication was repeated when periclinal and outer anticlinal walls were not totally removed with the treatment. Micromorphology was examined under scanning electron microscopy (SEM) after gold coating with a Hitachi S3000-N electron microscope at ×1200 magnification. Lateral and overhead images were taken of representative epidermal cells from each sample (Fig 2). Eight different measurements were taken directly from the photomicrographs. To minimize perspective and scale effects in lateral pictures, micromorphological characters were included as five different ratios, scaling heights by widths, therefore coding shape parameters (Table 3). Only igw was directly included as an average measurement, since it was obtained from the overhead picture and scale was expected to be constant. Being aware of differences in maximum width among epidermis cells of the same individual, we selected and measured the largest cells in each sample. The resulting micromorphological dataset was analyzed using PCA (mPCA). Quartile distribution for the macromorphological study was also calculated. All statistical analyses were performed in PAWS Statistics 18 as explained above.

### Distribution modeling

Species distribution modeling (SDM) was used to evaluate the climatic niche and potential distribution of population complexes within the bent-beaked clade of the *C*. *flava* group. We analysed four population/species datasets: the two dwarf morphotypes confined to different mountain systems (Alps, Pyrenees-Cantabrian Range), and *C*. *flava* and *C*. *lepidocarpa*, the two widely distributed well-developed large morphotypes. The Atlas and Sierra Nevada population sets were not included in the analyses because of the low numbers of localities available (7 and 11, respectively). It must be noted that even though the dwarf morphotypes seem to be linked to mountain environments, the distinction between well-developed large and dwarf morphotypes is based on morphology, and not on altitude thresholds. Indeed, populations of large morphotypes have been found at altitudes as high as ~2000 m in the Alps (e.g. *C*. *flava* in Risoul; *C*. *lepidocarpa* in Col du Lautaret; see S2 Appendix).

For modeling analyses, we employed the maximum entropy algorithm, as implemented in Maxent v.3.3 [57], because it is appropriate for presence-only data and its good predictive performance has been demonstrated [53]. We retrieved a set of 19 bioclimatic variables at 30 seconds resolution under current conditions from the WorldClim website (www.worldclim.org; [58]). Layers were clipped to the extent of Europe and the Mediterranean region. To test for multicollinearity of variables, pairwise Pearson’s correlation coefficients were calculated for a random sample of 1000 points of the study area generated using Hawth’s Analysis Tools for ArcGIS [59]. We did not find any high correlation (*r*≥0.70; S1 Table) among variables and therefore all 19 bioclimatic variables were included in the Maxent models. In the occurrence dataset, we included a total of 361 point localities (Fig 1), including 99 of *C*. *flava*, 118 of *C*. *lepidocarpa*, 59 of the dwarf morphotype from the Alps and 67 of the dwarf morphotype from Pyrenees-Cantabrian Range. These four sets of localities were modelled separately. We extracted occurrence information of each population group from herbarium specimens from ARAN, BM, GAP, JACA, FI, LEB, M, MA, MPU, MSB, NEU, P, RNG, SOM and UPOS. We also included localities listed in [60,61], which were considered taxonomically reliable sources. In the case of *C*. *flava* and *C*. *lepidocarpa*, we focused on the regions geographically close to the dwarf morphotypes, in order to accurately detect differences in niche requirements at a fine scale. Localities were geo-referenced using Google Earth, and only those with a precision higher than ± 4 km were selected. This precision represented a balance between the inclusion of a small number of highly accurate localities, and the inclusion of a large number of inaccurate localities. Localities are listed in the S1 Appendix. When running the analysis, each occurrence dataset was randomly split into training data (80%), used for model building, and test data (remaining 20%), used to evaluate model accuracy. Ten subsample replicates were performed of each analysis, and fitness of the resulting models was assessed with the area under the receiver-operating characteristic (ROC) curve [57]. A jackknife analysis was employed to evaluate variable contributions to the models. To convert continuous suitability values to presence/absence, we chose the threshold obtained under the maximum training sensitivity plus specificity rule, which has been shown to produce accurate predictions [62].

We assesed the climatic niche overlap between the well-developed large and dwarf mountain morphotypes using environmental-space (E-space) analyses based on Schoener’s D values [63]. We evaluated overlap at two scales: (1) pairwise comparisons between the entire areas of considered groups; and (2) parwise comparisons at regional scale between areas where the morphotypes co-occur (Alps and Pyrenees-Cantabrian Range; Fig 1). The E-space was represented in a Principal Component Analysis (PCA) as implemented in the R package *ecospat* [64]. Niche overlap between morphotypes was represented by plotting together the E-space of each pair of morphotypes. We also performed tests of niche equivalency and niche similarity, which compare the observed niche overlap with the overlap obtained using random subsets of samples [65,66], as implemented in *ecospat* too [64]. As we were studying very closely related sets of populations, we explored niche conservation (the niche overlap is more equivalent/similar than expected by chance) between each compared pair of morphotypes using the option “*greater”*. The niche equivalency test determines whether the observed niche overlap is constant when randomly reallocating the occurrences of both entities between their ranges. On the other hand, the niche similarity test checks whether the overlap between two niches is more similar than the overlap obtained if random shifts within each environmental space are allowed. When comparing the niche of a dwarf morphotype with that of *C*. *flava* or *C*. *lepidocarpa*, we set the dwarf morphotype niche as reference, and only allowed the well-developed morphotype niche to randomize (rand.type = 2). This allows testing the effect of shifts from the ancestral well-developed morphotype (see Results) into the derived dwarf morphotype’s space [64]. When we compared between dwarf morphotypes, or between well-developed morphotypes, we allowed random shifts between the two areas (rand.type = 1). All tests were based on 100 iterations.

### Phylogenetic analyses

A total of 31 individuals were sequenced for ITS, *rps*16 and 5’*trn*K (S1 Appendix). Most sequences (24 individuals) were taken from a previous phylogenetic study [18]. Sampling was expanded by obtaining ITS, *rps16* and 5´*trn*K sequences from seven additional individuals, mainly of populations from the Alps (S1 Appendix). The sampling consisted of 20 individuals from different populations of the four groups of dwarf morphotypes of sect. *Ceratocytis* and 11 individuals unequivocally ascribed to well-developed large morphotypes (*C*. *demissa* subsp. *demissa*, *C*. *demissa* subsp. *cedercreutzii*, *C*. *flava*, *C*. *hostiana*, *C*. *lepidocarpa* subsp. *lepidocarpa*, *C*. *lepidocarpa* subsp. *jemtlandica* and *C*. *viridula*). The well-defined taxa were chosen to represent the molecular variation of sect. *Ceratocytis* in Europe and North Africa detected in our previous study [18]. *Carex castroviejoi* was excluded due to its incongruent placement in nuclear and plastid phylogenies to avoid this conflicting signal, although this does not affect the topological relationships among the groups of populations studied here. *Carex cretica* was selected as outgroup in accordance with our previous reconstruction [18]. Herbarium specimens (M, MA) and silica-dried field-collected materials (vouchers deposited at UPOS) were included. Destructive sampling permission for DNA extraction was provided by these institutions. Total DNA was extracted using the DNeasy Plant Mini Kit (Qiagen, California). The PCR conditions and primers followed [18]. Sequencing was carried out by Stab Vida (Caparica, Lisboa, Portugal). Sequences were edited using Seqed (Applied Biosystems, California). Only one informative indel was found and coded manually as an additional binary character. Bayesian phylogenetic analyses were conducted with MrBayes v.3.2 [67]. The model of sequence evolution that best fits the data was selected using the Akaike information criterion (AIC) in jModelTest [68]. Models were calculated independently for each plastid marker, and also for each ITS region (ITS1, 5.8S and ITS2). The indel was analysed under the F81 model [67]. Two parallel Markov Chain Monte Carlo were run for 10,000,000 generations with a sampling interval of 1000 generations. We applied a burn-in of 25% to ensure stationarity after checking with Tracer [69]. The remaining trees were summarized in a majority rule consensus tree, with posterior probability (pp) as the measure of clade support. We also performed a maximum likelihood (ML) analysis as implemented in RAxML 8 [70] to complement the Bayesian analysis. The analysis was partitioned and the coded indel was maintained as a binary character. One hundred non-parametric bootstrap replicates were performed to assess topology uncertainty.

### Ancestral morphotype reconstruction

To analyze morphotype shifts in the course of diversification of the *C*. *flava* group, and to find out if the shared morphological traits are the result of convergent evolution, we reconstructed morphological ancestral states in Mesquite v.3.0 [71]. We used trees obtained from the Bayesian phylogenetic analysis described above. Given the lack of clearly defined operational taxonomic units (OTUs) within the bent-beaked clade [18], we decided to select samples for the ingroup following these criteria: (1) two sets of morphotypes were considered, the well-developed large morphotypes (*C*. *flava* and *C*. *lepidocarpa*) and the dwarf morphotypes; and (2) for each morphotype we kept one sample per detected plastid haplotype. In this way, we representatively covered the detected genetic variation in each set of morphotypes, and minimized the random overweight of a particular morphotype over the others. Outside the ingroup, only one sequence per species was kept. All other samples were pruned from the Bayesian posterior distribution of phylogenetic trees prior to the reconstruction using Mesquite.

Morphotype was coded as a qualitative trait with two character states: “well-developed large” (large plants, with all the reproductive diagnostic characters conspicuousy developed), and “dwarf” (small plants, with the reproductive characters reduced). The analysis was performed using the parsimony reconstruction method. In order to account for the uncertainty in tree topology, all pruned trees from the stable Bayesian posterior distribution were analyzed using the “Trace character over trees” option in Mesquite.

### Nomenclatural Acts

The electronic version of this article in Portable Document Format (PDF) in a work with an ISSN will represent a published work according to the International Code of Nomenclature for algae, fungi, and plants, and hence the new names contained in the electronic publication of this PLOS ONE article are effectively published under that Code from the electronic edition alone, so there is no longer any need to provide printed copies.

In addition, new names contained in this work have been submitted to IPNI, from where they will be made available to the Global Names Index. The IPNI LSIDs can be resolved and the associated information viewed through any standard web browser by appending the LSID contained in this publication to the prefix http://ipni.org/. The online version of this work is archived and available from the following digital repositories: PubMed Central, LOCKSS.

## Results

### Macromorphological variation

**Fig 3:**
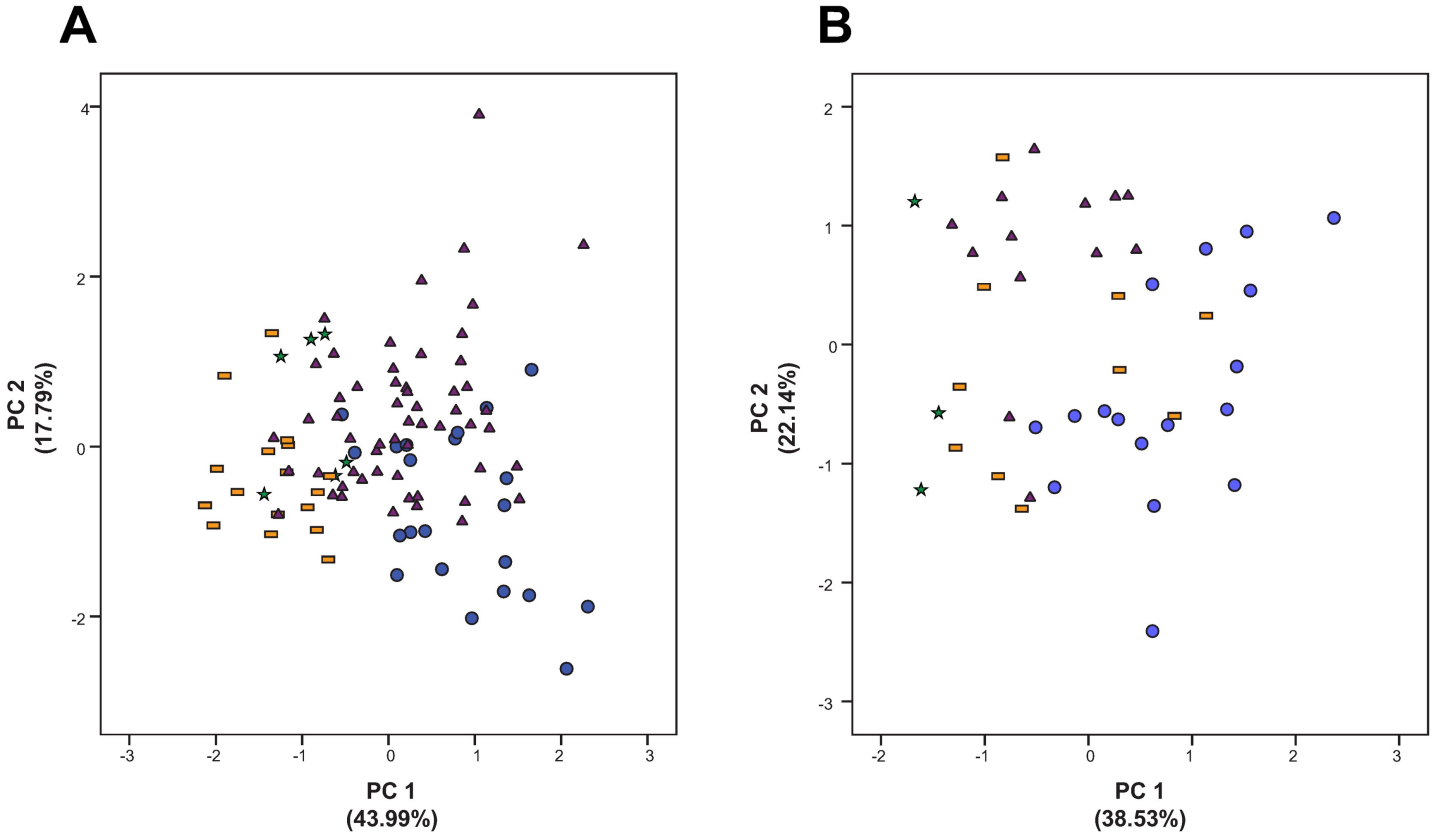
Scatter plots from the multivariate analyses based on macromorphological (A) and micromorphological (B) data of the dwarf morphotypes of the *Carex flava* group in Circum-Mediterranean mountains. Each morphotype is depicted with a different symbol and colors as in Fig 1: Alps (blue circles), Pyrenees-Cantabrian Range (purple triangles), Sierra Nevada (yellow rectangles) and Atlas (green stars). Contribution to the total variance by each component is shown next to the axis.

**Fig 4:**
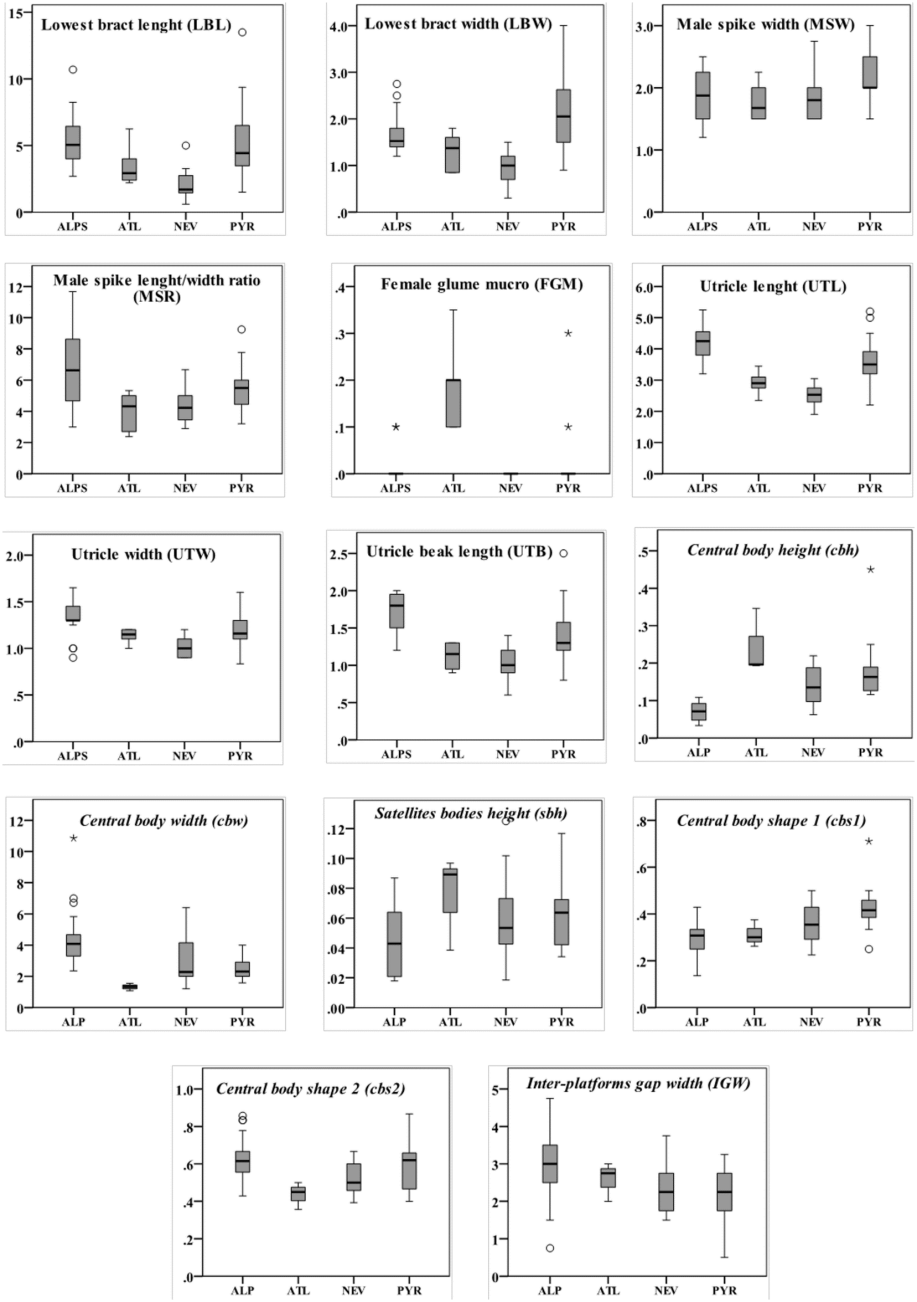
Box plots representing the studied characters and their variation in the four different sets of dwarf morphotypes. Morphotypes are abbreviated as follows: Alps (ALPS), Atlas (ATL), Sierra Nevada (NEV) and Pyrenees-Cantabrian Range (PYR). Macromorphological characters are given in regular font and measured in mm. Micromorphological characters are given in italics and measured as ratios. Characters are abbreviated according to Table 3.

The first two components showed eigenvalues higher than one. They accounted for 61.78% of the total variance (43.99% for PC-1; 17.79% for PC-2) in the dataset. Examination of the scatter-plot (Fig 3A) from these principal components revealed the partial overlapping of all dwarf mountain morphotypes. Plants from the Alps were displaced toward the highest scores of PC-1, whereas those from the Atlas and Sierra Nevada appeared toward the lowest values. Specimens from the Pyrenees-Cantabrian Range were displaced towards the highest values of PC-2. Morphological characters with the highest loadings were, in descending order, utricle length and utricle beak lenght for PC-1, and male spike width and lowest bract width for PC-2 (Table 3). Characters displaying less than a 25% overlapping threshold are displayed in Table 4 and Fig 4. The main diagnostic macromorphological characters that allow distinction among dwarf morphotypes are summarized in Table 5.

**Table 4:** Comparisons among the *Carex flava* group dwarf morphotypes from different mountain ranges based on morphological data. Characters listed display less than 25% overlap between dwarf morphotypes. Character abbreviations as in Table 3.

	**Alps**	**Atlas**	**Pyrenees-Cantabrian Range**
**Atlas**	FGM UTL UTB UTW		
	cbh cbw cbs2		
**Pyrenees-Cantabrian Range**	-	FGM UTL	
	cbh cbw cbs1	cbw cbs1 cbs2	
**Sierra Nevada**	LBL LBW UTL UTW UTB	FGM	LBL LBW UTL UTW UTB
		cbh cbw	

**Table 5:** Summary of the most important macro and micromorphological characters within dwarf mountain morphotypes of the *Carex flava* group.

Mountain Range	Macromorphology		Micromorphology	
	Inflorescence and spikes	Utricles	Central body	Satellite bodies
**Alps**	Lowest bract 30.0–91.8(110.0) × 1–2.5(3), leaf-like. Male spike 1–2.7(3.0) mm width, fusiform. Female glumes brown, muticous.	(3.0)3.1–5 × 1–1.7(2) mm, with a (1)1.2–2.2 beak, generally yellowish.	Flattened, scarcely sharp, filling an area no more than a quarter of the platform surface; sometimes inconspicuous.	Scarcely developed, flattened, sometimes a bit prominent and sharp.
**Pyrenees-Cantabrian Ranges**	Lowest bract 1.5–9.9(13.5) × 0.9–3.7(4), leaf-like, rarely setaceous. Male spike 1.5–2.9(3) mm width, fusiform. Female glumes light to dark brown, muticous, rarely with a mucro up to 0.3 mm.	(2.2)2.4–4.5(5.2) × 0.8–1.5(1.6) mm, with a 0.8–1.9(2.5) beak, greenish to yellowish or light brownish.	Well-developed, sharp to rounded, filling an area of approximately half of the platform surface.	Well-developed, prominent, rounded.
**Sierra Nevada**	Lowest bract 1–5.1(13) mm, setaceous, rarely lowermost spike arising from a lower leaf axil. Male spike 1.5–2.5(3) mm wide, fusiform to narrowly eliptical. Female glumes dark brown, muticous.	2–3 × 1 mm, with a 0.9–1.2 mm beak, dark brownish at least in the distal part.	Flattened but sharp, filling an area no more than a quarter of the platform surface.	Well-developed, prominent, rounded.
**Atlas**	Lowest bract (10)22.0–62.5 × 0.8–1.8 mm, leaf-like to setaceous. Male spike 1.5–2.2 mm width, fusiform to narrowly elliptical. Female glumes light brown to almost hyaline, with a 0.1–0.3 mm mucro.	2.3–3.4 × 0.8–1.8 mm, with a 0.9–1.3 beak, greenish to yellowish.	Well-developed, sharp to rounded, filling an area of at least half of the platform surface.	More or less well-developed, not too prominent, rounded.

### Micromorphological variation

SEM photographs revealed that the general features of the studied samples agree with previous studies on sect. *Ceratocystis* [45]. The inner anticlinal wall of the epidermic cells bears a large central ± conical silica body elevated on a narrower platform that is surrounded by a variable number of peripheral smaller silica bodies. The morphometric analyses found slightly different variation patterns among the dwarf morphotypes (Fig 3). The first two components showed eigenvalues higher than one. Principal component analysis of the micromorphological dataset (mPCA; Fig 3B) did not differentiate populations from different mountain ranges. Samples from Atlas plants were recovered in the periphery of the scatter-plot, displaced towards the lowest values of PC-1. Those from the Alps were placed along PC-2, showing relatively high PC-1 scores, whereas the samples from the Pyrenees-Cantabrian Range were located at lower values of PC-1. The specimens from Sierra Nevada were intermingled among the samples from the Alps and Pyrenees-Cantabrian Range. The first two components accounted for a total variance of 60.67% (38.53% for PC-1; 22.14% for PC-2). Micromorphological characters with the highest loadings for each component were central body height and central body width for PC-1, and central body shape 1 and central body shape 2 for PC-2 (Table 3). Characters overlapping equal or below 25% in pairwise comparisons between dwarf morphotype groups are displayed in Table 4 and Fig 4. The main diagnostic micromorphological characters are summarized in Table 4.

### Distribution modeling

**Fig 5:**
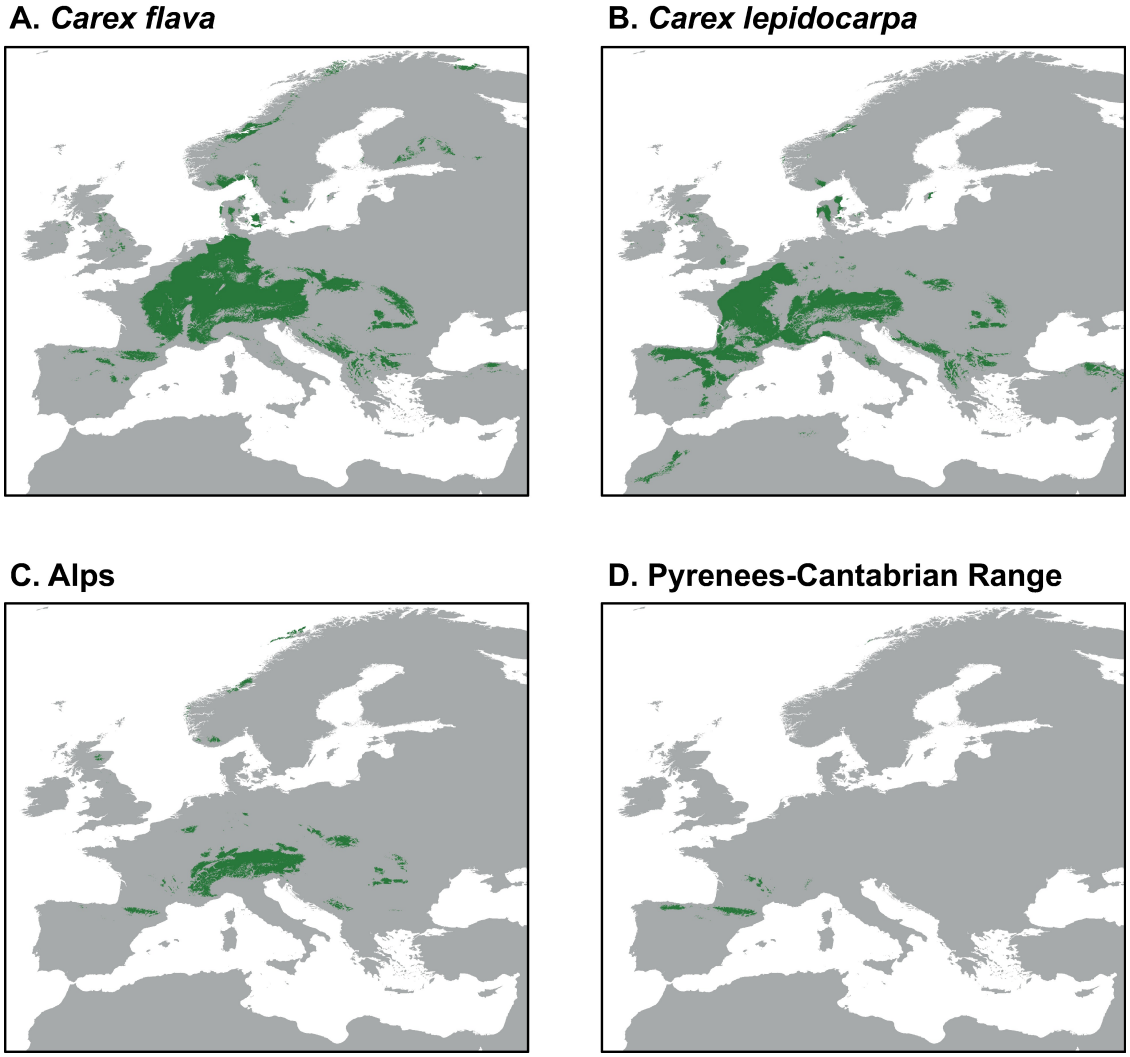
Distribution modeling of morphotypes belonging to the bent-beaked clade of the *Carex flava* group. The average distribution models fitted to current climatic conditions are shown for the well-developed individuals (A, *C*. *flava*; B, *C*. *lepidocarpa*) and dwarf morphotypes (C, Alps morphotype; D, Pyrenees-Cantabrian Range morphotype).

The average distribution models (Fig 5) supported differences in potential distribution between the well-developed large and dwarf morphotypes. High values of the area under the ROC curve (between 0.9 and 1.0) were obtained. *Carex flava* and *C*. *lepidocarpa* displayed similarly widespread potential distributions mainly in Central Europe, where they spanned lowland and montane (below timberline) areas. Suitable areas were also detected in the Mediterranean peninsulas (Iberian, Italian, Balkan and Anatolian), but restricted to mountain areas. According to jackknife tests, the most informative variables for the models were bio18 (precipitation of warmest quarter) and bio17 (precipitation of driest quarter) for *C*. *flava* and *C*. *lepidocarpa*, respectively.

For the dwarf mountain morphotypes, we inferred more restricted potential distributions mainly in southern European mountains: Alps (mainly for the Alps population set), Pyrenees (for both the Pyrenees-Cantabrian Range and Alps sets), and Cantabrian Range (mainly for the Pyrenees-Cantabrian Range set). Sierra Nevada and the Atlas were not recovered as part of the potential ranges of either the Alps or the Pyrenees-Cantabrian Range population sets. The potential areas of well-developed large and dwarf morphotypes overlapped at mid-altitudes in the Alps and Pyrenees. However, higher altitudes were inferred as only suitable for the dwarf morphotypes, while the adjacent lowlands were inferred as only suitable for the well-developed large morphotypes (Supplementary S1 Fig).

**Fig 6:**
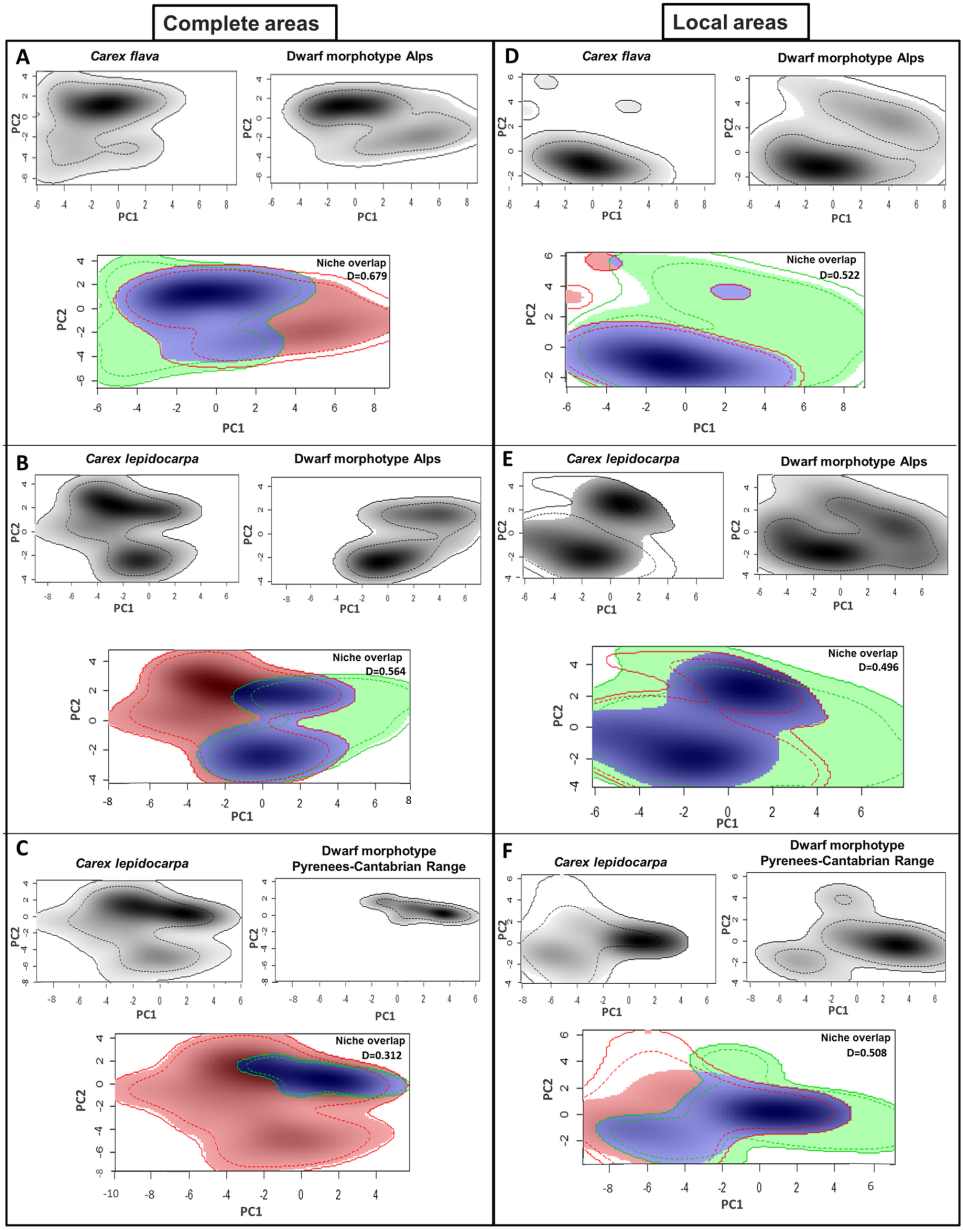
Principal component analyses (PCA-env) for pairwise morphotype comparisons in the climatic space, using the whole distribution ranges (A-C) and the regional co-ocurring areas (D-F). For each comparison, the two upper plots separately represent the respective niches of the well-developed/dwarf morphotype along the first two axes of the PCA, while the lower plot represents the niches jointly. The solid and dashed lines show 100% and 50%, respectively, of the available environment. Green and red lines/shapes indicate the respective climatic space of each compared morphotype and the blue shape the overlapping area. Gray shading displays the density of occurrences of the morphotypes by cell.

PCA plots showing niche overlap between well-developed and dwarf morphotypes in E-space are displayed in Fig 6. The contributions of the set of bioclimatic variables to the axes of the different PCAs are included in S2 Fig. Pairwise statistical tests for comparison of ecological niches and Schoener’s D values are shown in Table 6. The largest value of Schoener’s D was obtained for the comparison of the whole ranges of well-developed morphotypes of *C*. *flava* and *C*. *lepidocarpa* (Fig 6, Table 6). On the contrary, the lowest value of D was obtained for the niche overlap between the two dwarf morphotypes. D values also revealed that when the whole ranges are tested, both well-developed morphotypes overlap more with the dwarf morphotype from the Alps than with the dwarf morphotype from Pyrenees-Cantabrian Range. Remarkably, comparisons of regional co-occurring areas display similar D values between well-developed morphotypes and the co-ocurring dwarf morphotypes (Table 6). The niche similarity and equivalency tests revealed that, when comparing the entire areas of well-developed and dwarf morphotypes, these are significantly more similar than expected by chance, but not identical. The comparison between *C*. *flava* and *C*. *lepidocarpa* using their entire areas revealed that their niches were both more similar and more identical than expected at random. On the contrary, the comparison of the two dwarf morphotypes (Alps vs. Pyrenees-Cantabrian Range) retrieved that their niches are well differentiated, not meeting similarity or equivalency (Table 6). When the comparisons were restricted to populations co-occurring in the Alps or the Pyrenees-Cantabrian Range, the results were not significant for any of the pairwise comparisons, revealing differences between the niches of both co-ocurring well-developed morphotypes and the dwarf morphotypes, but also between populations of well-developed *C*. *flava* and *C*. *lepidocarpa* from the Alps (Table 6).

**Table 6:** Pairwise statistical tests for comparison of ecological niche overlap, niche equivalency, and niche similarity between different sets of populations of well-defined and dwarf morphotypes. In each case, the hypotheses tested were whether the niches are more equivalent/similar than expected by chance. Statistical significance is represented by p-values, and marked with an asterisk (*) when significant (p<0.05). rt1 = rand.type1, both ranges were allowed to shift; rt2 = rand.type2, the dwarf morphotype range was fixed, and only the range of the well-developed morphotype was allowed to shift (see Materials and methods for details).

	Numbers of populations (N)	Schoener’s D	Niche equivalency	Niche similarity
**Comparisons of whole ranges**				
*C*. *flava* vs. *C*. *lepidocarpa*	99/118	0.761	0.01*	rt1 = 0.01*
*C*. *flava* vs. dwarf morphotype Alps	99/59	0.679	0.30	rt2 = 0.01*
*C*. *flava* vs. dwarf morphotype Pyrenees-Cantabrian Range	99/67	0.269	1	rt2 = 0.01*
*C*. *lepidocarpa* vs. dwarf morphotype Alps	59/118	0.564	0.55	rt2 = 0.01*
*C*. *lepidocarpa* vs. dwarf morphotype Pyrenees-Cantabrian Range	118/67	0.312	1	rt2 = 0.01*
Dwarf morphotype Alps vs. Dwarf morphotype Pyrenees-Cantabrian Range	59/67	0.199	1	rt1 = 0.31
**Comparisons of regional co-occurring areas**				
*C*. *flava* (Alps) vs. *C*. *lepidocarpa* (Alps)	54/36	0.466	0.94	rt1 = 0.19
*C*. *flava* (Alps) vs dwarf morphotype Alps	54/59	0.522	1	rt2 = 0.06
*C*. *lepidocarpa* (Alps) vs. dwarf morphotype Alps	36/59	0.496	0.70	rt2 = 0.13
*C*. *lepidocarpa* (Pyrenees-Cantabrian Range) vs. dwarf morphotype Pyrenees-Cantabrian Range	26/67	0.508	0.782	rt2 = 0.16

### Phylogenetic analyses

**Fig 7:**
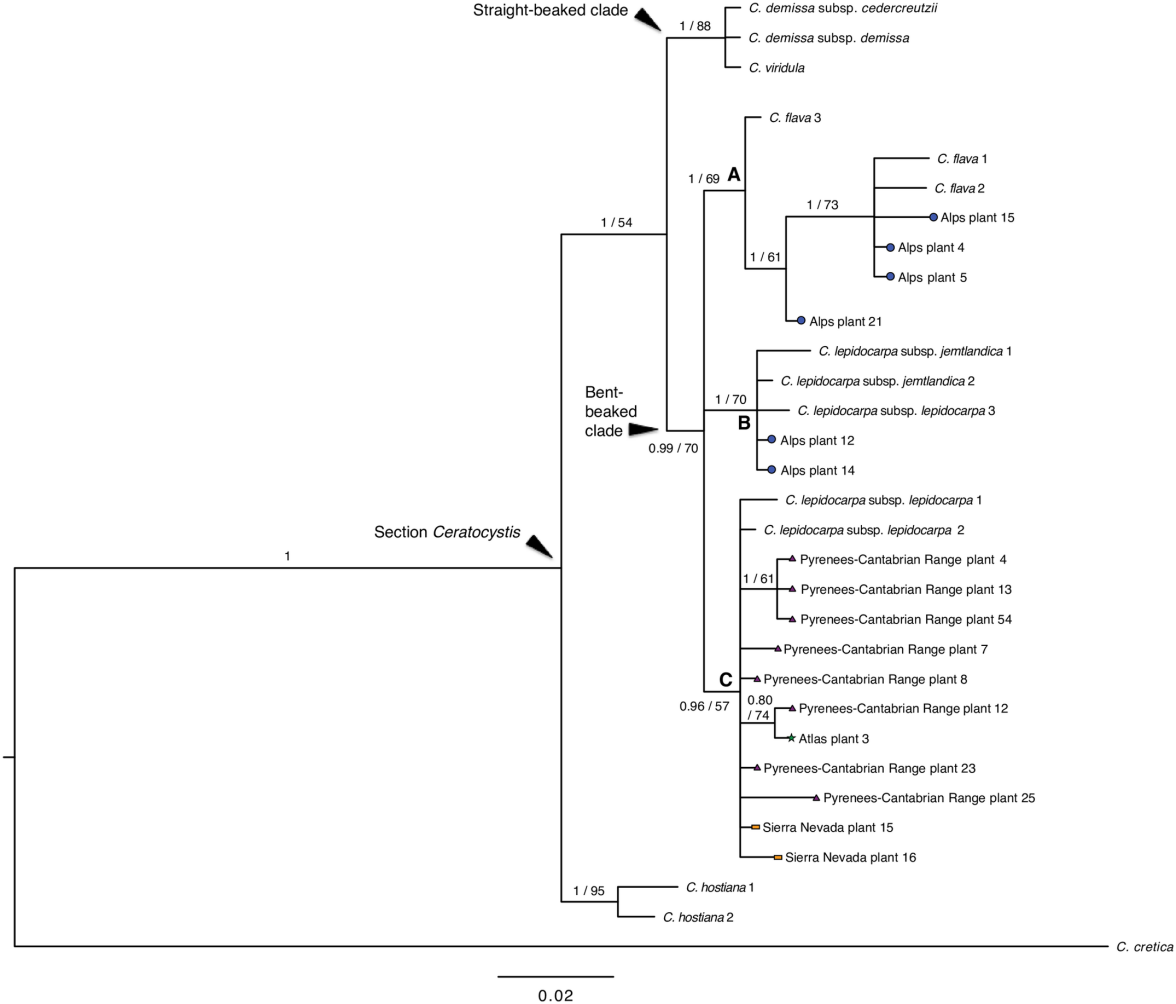
Fifty-percent majority rule consensus tree obtained from the Bayesian phylogenetic analysis of *Carex* sect. *Ceratocytis* based on the combined matrix of ITS and *rps*16-5’*trn*K sequences with coded indels. Bayesian posterior probabilities equal or greater than 0.8 are given next to the branches before the slash; bootstrap supports from the ML analysis are given after the slash when greater than 50. Straight-beaked and bent-beaked clades are indicated. Well-supported clades within the bent-beaked clade are named as follows: (A) *C*. *flava* lineage, (B) *C*. *lepidocarpa* B lineage, and (C) *C*. *lepidocarpa* C lineage. Sample numeration follows S1 Appendix. Shapes at the terminals indicate the dwarf mountain morphotypes according to Figs 1 and 3.

Models selected for each DNA region were GTR+I for ITS-1, *rps*16 and 5’*trn*K, K80 for 5.8S, and GTR+G for ITS-2. Our Bayesian phylogenetic reconstruction using the concatenated matrix with the three markers (ITS-*rps*16-5’*trn*K) yielded a consensus topology (Fig 7) congruent with that found in [18]. The ML topology agreed with the Bayesian tree but with overall lower support values (Fig 7). The sect. *Ceratocystis* was divided in three main strongly supported clades: the *C*. *hostiana* clade (1 pp; 95 bs), sister to the other two clades; the straight-beaked clade (1 pp; 88 bs), which includes *C*. *demissa* and *C*. *viridula*; and the bent-beaked clade (0.99 pp; 70 bs) with all the remaining samples. Within the bent-beaked clade, three main well-supported subclades (A-C) were found, each containing a different set of well-defined taxa and dwarf morphotypes. Subclade A (1 pp; 69 bs) contained *C*. *flava* and the mountain morphotype from western and central Alps (hereafter *C*. *flava* lineage). Subclade B (1 pp; 70 bs) contained *C*. *lepidocarpa* subsp. *jemtlandica*, an eastern European *C*. *lepidocarpa* subsp. *lepidocarpa* population, and the mountain morphotype from eastern Alps (hereafter lineage *C*. *lepidocarpa* B). Subclade C (0.96 pp; 57 bs) grouped Iberian *C*. *lepidocarpa* subsp. *lepidocarpa* populations with the mountain morphotypes from Sierra Nevada, Pyrenees-Cantabrian Range, and the only sampled population from the Atlas (hereafter lineage *C*. *lepidocarpa* C).

### Ancestral morphotype reconstruction

**Fig 8:**
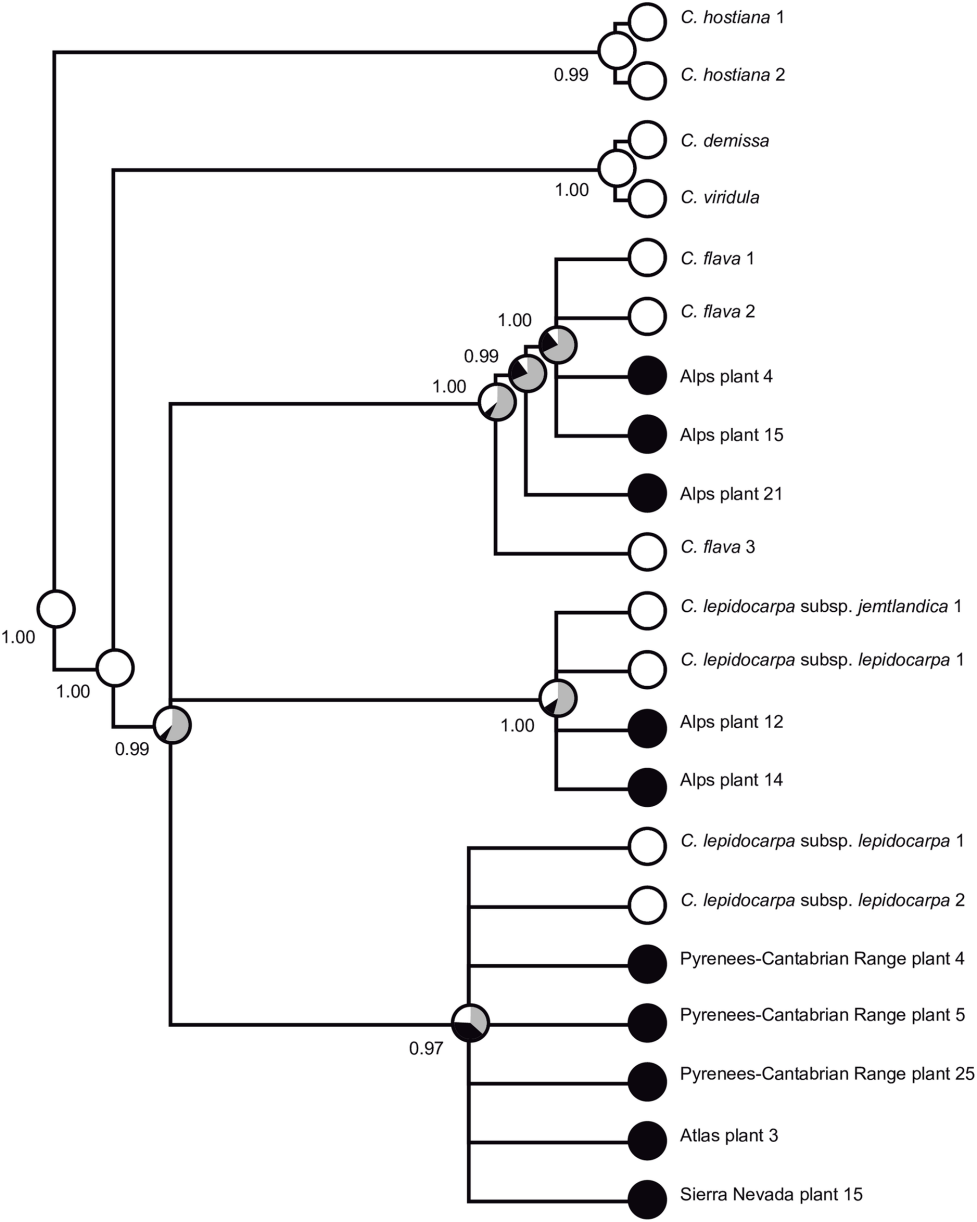
Ancestral state reconstruction of morphotypes in *Carex* sect. *Ceratocytis*. Results are summarized on the 50% majority rule consensus tree obtained from the Bayesian phylogenetic analysis. Pie charts at nodes summarize the results of the parsimony optimization conducted over the posterior distribution of trees from the Bayesian analysis. Each chart shows the proportion of trees for which a given morphotype was reconstructed for that node: dwarf (black), well-developed (white), and equivocal (gray). Terminals were pruned to leave only one representative per morphotype and haplotype, as explained in Materials and Methods.

Ancestral morphotype was unambiguously inferred as “well-developed” (100% of trees) for the most recent common ancestor (MRCA) of *Carex* sect. *Ceratocystis* and also for the MRCA of the bent-beaked and straight-beaked clades, and that of the straight-beaked clade (*C*. *demissa* and *C*. *viridula*) (Fig 8). However, the ancestral morphotype was mostly equivocal (>50% of trees) for the MRCAs of the bent-beaked clade, *C*. *flava* (subclade A) and *C*. *lepidocarpa* B lineage (subclade B). For the *C*. *lepidocarpa* C lineage (subclade C), we obtained similar proportions of “dwarf” morphotype and equivocal reconstructions. Excluding the equivocal reconstructions, the most probable ancestral morphotype was inferred as “well-developed” for the whole bent-beaked clade and the *C*. *flava* and *C*. *lepidocarpa* B lineages, and “dwarf” for the *C*. *lepidocarpa* C lineage.

## Discussion

### Cut from the same cloth: Morphological convergence, a result of adaptation to mountain environments?

Previous molecular phylogenetic analyses [18] already showed that different dwarf morphotypes had taxonomic affinities with at least two different lineages, suggesting their independent origins. Accordingly, our molecular data revealed that mountain populations of the *C*. *flava* group are genetically heterogeneous and belong to three different lineages. Macromorphological studies not focused on the dwarf mountain plants have shown that differences between species, when tested in well-developed plants, are taxonomically significant [21,23,26,27,72]. However, our macromorphological study focused on dwarf mountain plants show that the differences between these populations are subtle or inexistent. This result explains previous proposals that mountain dwarf morphotypes constitute a single taxon [30,31] despite the molecular evidence to the contrary. The close resemblance of morphotypes from the Alps and the Pyrenees-Cantabrian Range is especially striking. Even though populations belong to three different lineages (Fig 7), they show a wide degree of overlap in the MPCA (Fig 3A), with no macromorphological characters showing an overlap below 25% (Table 4). The phylogenetic relationships of the Alps and Pyrenees-Cantabrian Range dwarf morphotypes (Fig 7), together with their lack of macro-morphological differentiation (Fig 3A; Table 4) and inferred history of morphological evolution (Fig 8), point to convergent evolution as the process behind their morphological resemblance. The dwarf morphotypes do not form a monophyletic group and are recovered within different lineages (Fig 7). Despite the uncertainty on ancestral state reconstruction (Fig 8), the morphotype with the highest probability was “well-developed” for the MRCA of the bent-beaked clade, *C*. *flava* and *C*. *lepidocarpa* B lineages. We interpret these results as suggesting the convergent acquisition of the dwarf morphotype by certain mountain populations of these two species.

Morphological convergence induced by similarly harsh environmental conditions is frequently found in mountain plants (Table 1), and sometimes produces identical adaptational responses in different groups [8,11,15], even resulting in adaptative radiations [9,13]. The selective pressures in mountain habitats seem to modify the well-developed large growth forms found in lowland environments, leading to similar morphologies regardless of the ancestral genotype (cf. [1]). The results of niche overlap, equivalency and similarity indeed point in this direction. The niche similarity but not equivalence between well-defined morphotypes and dwarf-morphotypes when tested using the whole ranges (Table 6) indicates that all of them are closely related plants that share ancestral ecological features, but also display underlying differences [73]. When the comparisons were restricted to well-defined and dwarf morphotypes co-occurring in a same mountain range, there is an overall lack of similarity and equivalence (Table 6), indicating that their regional niche spaces are not interchangeable. This is consistent with a role of ecological differentiation as a driver of morphological differentiation. The potential distributions inferred by our modeling analyses for dwarf populations indeed retrieve higher-altitude habitats that are colder and more exposed than those preferred by their lowland, paraphyletic counterparts (S1 Fig; see also [29]), which suggests the dwarf habit as an adaptation to the harsh conditions including strong winds of high mountains.

The effect of the geographic restriction on the significance of the comparisons further points to the role of mountain environments in the acquisition of dwarf morphotypes. Niche similarity between well-developed and dwarf morphotypes was only found when tested over the entire Euro-Mediterranean region, but not when the analyses were restricted to a particular mountain range. This implies that certain populations of well-developed morphotypes inhabiting non-mountainous areas are experiencing bioclimatic conditions that are to some extent similar to those of the dwarf morphotypes inhabiting mountain ranges. However, the adaptative response of these well-developed plants did not entail the evolution of a dwarf morphotype.

The potential areas of the Alps and Pyrenees-Cantabrian Range dwarf population sets partially overlap, mainly in the Pyrenees. In contrast, very small areas of the Cantabrian Range are recovered as potential habitat for the Alps set, and very small areas of the Alps are recovered as potential habitat for the Pyrenees-Cantabrian Range set (Fig 5C and 5D). Accordingly, the equivalency and similarity tests did not reveal significance for the comparison between dwarf morphotypes from different areas (Table 6). These differences may be the result of the slightly different climatic conditions of these mountain ranges.

### Incipient divergence: Subtle differences in the southernmost populations as a possible result of isolation

The populations from Sierra Nevada and the Atlas, which are the most isolated of the complex, show a certain degree of morphological differentiation from the other dwarf morphotypes. A previous morphological exploration comparing dwarf and well-developed morphotypes already reported the morphological distinctiveness of the plants from Sierra Nevada and the Atlas. Such differentiation contrasted with the clinal variation found between well-developed large lowland morphotypes of *C*. *lepidocarpa* and the dwarf mountain morphotype from the Pyrenees-Cantabrian Range [21]. Our study, including only dwarf morphotypes, shows that in the MPCA both population sets are slightly displaced towards the lowest scores of PC-1, forming the less dispersed set of samples (Fig 3A). In addition, for several macro- and micromorphological features (Fig 3A and 3B, Tables 4 and 6), the Sierra Nevada and Atlas plants showed less than 25% overlap with all other studied populations. This, together with the finding of diagnostic qualitative morphological features (utricle and glumes color: both dark brown in Sierra Nevada populations vs. glumes light-brown or hyaline and utricles greenish to yellowish in Atlas populations; Table 5), readily allows the morphological distinction of the Sierra Nevada and Atlas populations from each other, and also from the other dwarf morphotypes. In addition, the apparent absence of potential habitats in Sierra Nevada and the Atlas for the Alps and Pyrenees-Cantabrian Range population sets (Fig 5C and 5D) suggests that dwarf populations in these southern mountains could be adapted to climatic conditions that are somewhat different to those of their northern counterparts.

Sierra Nevada and Atlas populations are both nested within clade C of *C*. *lepidocarpa* (Figs 7 and 8). The isolated geographic placement of the Sierra Nevada and Atlas populations strongly suggests a pattern of post-glacial south-to-north disruption of genetic exchange following global warming [74,75]. This is the process that appears to be behind the incipient morphological divergence of these southernmost populations from related counterparts. The process of divergence following isolation could be in their earliest stages in the Sierra Nevada and Atlas populations, with incipient morphological differentiation, but no differences yet in DNA sequences of the selected barcoding markers.

### Taxonomic implications

In comparison with the relatively wide range of climatic conditions where the well-developed forms of *C*. *flava* and *C*. *lepidocarpa* may grow, there seems to be a relationship between the dwarf morphotypes of the *C*. *flava* group and the climatic conditions of the mountains they inhabit. These morphologically similar morphotypes have been interpreted as bridges of clinal variation among taxa [30,31], traditionally complicating the taxonomy of the group. Our analyses show that their depauperate morphology is probably related to their habitat, by means of recurrent convergent adaptation to high mountain environments.

As a taxonomic summary, we propose that populations of the dwarf morphotype from the Alps should be considered within the variation of *C*. *flava* or *C*. *lepidocarpa*, depending on the population (Table 2). Such taxonomic identity may be addressed only by means of genetic barcoding. Populations from the Pyrenees and Cantabrian Range should be considered within the variation of *C*. *lepidocarpa* (although they are known to have experienced some degree of introgression from *C*. *demissa* [18]). On the contrary, the morphogeographic compartmentalization of the Sierra Nevada and Atlas populations, together with their low phylogenetic differentiation (Fig 7), support a status of these population sets as infra-specific taxa within the same species [39]. Therefore, a separate subspecies rank appears to be suitable for them: *C*. *lepidocarpa* subsp. *nevadensis* in Sierra Nevada (see Table 2) and a new subspecies in the High Atlas, which is herein described (*C*. *lepidocarpa* subsp. *ferraria*, S3 Fig).

*Carex lepidocarpa* Tausch. subsp. *ferraria* Jim.-Mejías & Martín-Bravo, *subsp*. *nov*. [urn:lsid:ipni.org:names: 77173728–1]

Holotype—MOROCCO: High Atlas, Adrar-n-Oukaïmeden, vertiente N, borde pedregoso de arroyo, granitos, 31°11'09''N 7°51’19”W, 3000 m, 29 Jul 2006, *A*. *Herrero* et al., *AH3090* (MA-746566!; S3 Fig).

Diagnosis—A subspecies similar to *Carex lepidocarpa* subsp. *nevadensis* (Boiss. & Reut.) Luceño, from which it differs by the paler female glumes that bear an apical scabrid mucro (*vs*. dark brown and without mucro in subsp. *nevadensis*), and the utricles light green (*vs*. dark brown in subsp. *nevadensis*).

Description.—Plant tufted. Fertile stems 1.5–14(20) cm long, trigonous, smooth, erect or slightly curved. Leaves 1–3 mm wide, shorter than or as long as stems, flat; ligule short, rounded to subacute, hyaline; basal leaf sheaths inconspicuous, weak, light brown to purplish. Inflorescence with the uppermost spikes clustered at top of the inflorescence, sometimes with a shortly pedunculated basal spike arising from the axil of a leaf; lowest bract 2.2–6.2 cm × 0.8–1.8 mm, generally longer than inflorescence, short leaf-like, rarely bristle-like. Male spike 1, 4–9 × 1.5–2.2 mm, narrowly fusiform, with a 2–7 mm peduncle. Female spikes 1–3, the lowermost 5–7 mm long. Male glumes oval, subacute to obtuse, light brown, with a lighter middle nerve and a conspicuous hyaline margin. Female glumes oval, obtuse, mucronate, with a mucro 0.1–0.3 mm long, light brown to almost hyaline, with a lighter single middle nerve. Utricles 2.3–3.4 × 0.8–1.8 mm, greenish to yellowish, those from the proximal half of each spike deflexed, those from the medial part spreading, and the distal ones ascending, elliptical, trigonous, plurinerved, abruptly attenuated to a 0.9–1.3(1.5) mm beak, straight to deflexed (0–50° respect to utricle body), bidentate or bifid, smooth. Stigmas 3. Achenes 1–1.5 × 0.5–1 mm, obovoid, trigonous.

Phenology.—June–July.

Ecology.—Wet soils in high mountains, over igneous rocks and schists. 2000–3200 m.

Etymology.—From *ferrarius*, Latin for blacksmith, “herrero” in Spanish. The new subspecies is dedicated to our colleague and friend Dr. Alberto Herrero, from the Real Jardín Botánico de Madrid (Spain), specialist in the taxonomy of Mediterranean plants, and collector of the holotype material.

Distribution.—Morocco, SW High Atlas, known from Jbel Oukaïmedem, Jbel Angour, Jbel Gourza, Jbel Toubkal and Reraya valley.

Paratypes.—Morocco, High Atlas: in Atlantis Majoris monte Gourza supra oppidum Amismiz, 2700–2800 m, 29 Jul 1925, R. Maire (MPU!); in Atlantis Majoris Valle Reraya, environs d’Armound 2000 m, 19 Jul1926, R. Maire (K!, MPU!, P digital image!); in Atlantis Majoris Valle Reraya, convallis Ouenkrim, 2700–3100 m, 20 Jul 1924, R. Maire (MPU!); in Atlantis Majoris Valle Reraya, convallis Ouenkrim, 2780 m, 20 Jul 1924, R. Maire (MPU!, P digital image!); Reraya, hte vallée de l’acif Ouenkrim, 3000–3180 m, 24 Jul 1923, R. de Litardiere (MPU!); Reraya, hte vallée de l’acif Ouenkrim, 2850–3180 m, 24 Jul 1923, R. de Litardiere (MPU!); 72 Km S from Marrakech, Oukaïmedem, 31°12’N, 7° 51’W, 2619 m, 4 Jul 1987, S.L. Jury et al. (MA!, RNG!); Marrakech, Hoher Atlas, Umgebung von Oukaimedem und Berge S des Ortes, 31°11’N, 7°51’W, 2500–3000 m, 10 Aug 1999, D. Podlech 55321 (MSB!); Marrakech, Oukaïmedem, Jbel Angour, 31°10’28”N, 7°50’43”W, 2820 m, 15 Jun 2001, S.L. Jury et al. (RNG!).

The following identification key helps to identify all the taxa of *Carex* sect. *Ceratocystis* from the Western Palearctic according to [21,26–28,76–78], and data provided by this study. Terminology for the prophylls follows [79].

The reader must be aware that the mountain populations from Pyrenees, Cantabrian Range and the Alps are morphologically depauperate forms that can be morphologically undistinguishable without the aid of DNA sequences.

1. Female glumes with broad conspicuous hyaline margin; female spikes cylindric to oblong; rhizomes laxly cespitose … *C*. *hostiana* DC.

1. Female glumes without hyaline margin, or with it narrow; female spikes oblong to globose; rhizomes densely cespitose … (2)

2. Male spike 3–4.2 mm wide, widely fusiform to elliptical, sessile; utricle beak bent with respect to the utricle body, smooth; proximal utricles of each spike deflexed … *C*. *castroviejoi* Luceño & Jim.Mejías

2. Male spike usually <3 mm wide, linear or narrowly fusiform, sessile or pedunculate; utricle beak bent or straight, smooth or scabrid; proximal utricles of each spike spreading or deflexed … (3)

3. Utricles dark brown, at least in its distal half, up to 3 mm long; lowermost bract setaceous or shortly leaf-like, up to 1.5 mm wide … *C*. *lepidocarpa* subsp. *nevadensis* (Boiss & Reut.) Luceño

3. Utricles green, yellow, or light brown, 1.6–6(6.5) mm long; lowermost bract setaceous or leaf-like, to 1.5 mm or wider … (4)

4. Utricles from the middle portion of the female spikes clearly deflexed; utricle beak bent with respect to the utricle body (>25°) … (5)

4. Utricles from the middle portion of the female spikes spreading; utricle beak straight or bent with respect to the utricle body (usually <25°) … (6).

5. At least some utricles of the distal half of each spike with the beak conspicuously deflexed; male spike with a peduncle 2–40 mm; utricles 3.8–4.5(5) mm; lowermost bract shorter or up to 1.5× as long as the inflorescence … *C*. *lepidocarpa* Tausch. subsp. *lepidocarpa*

5. Utricle beaks of the distal half of all spikes spreading; male spike sessile or subsessile, very rarely with a peduncle up to 15 mm; utricles 4–6(7) mm; lowermost bract 2–5× as long as the inflorescence … *C*. *flava* L.

6. Utricle beaks from the proximal half of each spike deflexed; female spikes globose to ovate … (7)

6. Utricle beaks from the proximal half of each spike spreading, rarely slightly deflexed; female spikes ovate to oblong, rarely globose … (9)

7. Female glumes with a 0.1–0.2 mm scabrid mucro; utricles 2.3–3.4 mm long … *C*. *lepidocarpa* subsp. *ferraria* Jim.-Mejías & Martín-Bravo

7. Female glumes acute, without mucro; utricles (2.2)2.4–5.3 mm long … (8)

8. Beak of the proximal utricles of each spike conspicuously bent with respect to the utricle body; utricles (2.2.)2.4–5(5.2) mm long … Mountain dwarf forms of *C*. *flava* and *C*. *lepidocarpa*

8. Utricle beak straight; utricles 3.7–5.3 mm long … *C*. *lepidocarpa* subsp. *jemtlandica* Palmgr.

9. Female spikes crowded at the top of the inflorescence, the two distal-most ones separated by an internode 1–8(11) mm long; male spike sessile or with a 1–6.6(9) mm peduncle; utricles 2–3(4) mm long, with a 0.5–1.3 mm beak; leaves flat or canaliculate, up to 2.6(3) mm wide … *C*. *viridula* Michx. (including *C*. *derelicta* Štěpánková)

9. Female spikes frequently separated, the two distal-most ones separated by an internode 1.5–20(32) mm; male spike with a 1–10(21) mm peduncle; utricles 2.5–4(4.5) mm long, with a (0.6)0.7–1.8 mm beak; leaves flat, up to 3.7(5) mm wide … (10)

10. Utricles up to 1.5 mm wide; proximal utricles of each spike spreading to slightly deflexed; leaves equalling stems or up to 3(4) times shorter … *C*. *demissa* Hornem. subsp. *demissa* (including subsp. *iranica* Kukkonen)

10. Utricles up to 1.2 mm wide; proximal utricles of each spike spreading to slightly ascending; leaves 1.5–5 times shorter than stems … *C*. *demissa* subsp. *cedercreutzii* (Fagerstr.) Jac.Koopman

## Conclusions

The morphological resemblance among the mountain dwarf morphotypes of the *Carex flava* group seems to be at least partly the result of the recurrent convergent adaptation to harsh mountain environments in different lineages of the group. The subsequent underdevelopment of diagnostic morphological characters has contributed to this striking morphological resemblance of dwarf mountain plants, that led multiple authors to consider them as a single species different from all other taxa of the group.

## Supporting information

S1 Appendix Studied material. Letters or codes in brackets are indicated if samples were included in the macromorphological (*M*), micromorphological (*m*) or molecular study (ITS, *5’trnK* and *rps16* GenBank accession numbers); symbol * indicates new sequences obtained in this study; ×*n* indicates the number of samples included from the same population, if more than one was included.
(DOCX)

S2 Appendix Point localities employed in the distribution modeling analysis. Each tab of the spreadsheet displays localities of a well-developed or dwarf morphotype of the studied species. Information for each locality includes country, location, longitude and latitude in decimal degrees, collector and herbarium where the voucher is deposited, or reference if the information comes from a bibliographic source.
(XLSX)

S1 Fig Details of distribution models. Details of distribution models of well-developed individuals and dwarf morphotypes in the central Alps (A, B) and the central Pyrenees (C, D).
(TIF)

S2 Fig Relative contributions of the climatic variables to the principal components. Relative contributions of the climatic variables to the two axes of the PCAs. in Fig 6.
(TIF)

S3 Fig Holotype of *C*. *lepidocarpa* subsp. *ferraria* Jim.-Mejías & Martín-Bravo. *A*. *Herrero* et al., *AH3090*, MA 746566.
(TIF)

S1 Table Pairwise Pearson’s correlation coefficients. Pairwise Pearson’s correlation coefficients between the 19 WorldClim bioclimatic variables calculated for a random sample of 1000 points of the study area (Europe and the Mediterranean region).
(DOC)
