# Supplementary material for: Cut from the same cloth: The convergent evolution of dwarf morphotypes of the Carex flava group (Cyperaceae) in Circum-Mediterranean mountains
Source: PLoS One. 2017 Dec 27;12(12):e0189769. doi: 10.1371/journal.pone.0189769 (PMC5744957; doi:10.1371/journal.pone.0189769)
Supplement: S1 Appendix — Letters or codes in brackets are indicated if samples were included in the macromorphological (M), micromorphological (m) or molecular study (ITS, 5’trnK and rps16 GenBank accession numbers); symbol * indicates new sequences obtained in this study; ×n indicates the number of samples included from the same population, if more than one was included. (DOCX) [file pone.0189769.s001.docx]

**S1 Appendix.** Studied material. Letters or codes in brackets are indicated if samples were included in the macromorphological (*M*), micromorphological (*m*) or molecular study (ITS, *5’trnK* and *rps16* GenBank accession numbers); asterisks (*) indicate new sequences obtained in this study; ×*n* indicates the number of samples included from the same population, if more than one was included.

**Dwarf morphotype from the Alps** — **Austria 1.** Tal des Faltbaches S von Neuhaus am Zeilerrain *J. Poelt* et al., BM (*M*); **2.** Feuchten, Felsschutt, *M. Moore*, NEU (*M; m*); **3.** Oberosterreich, S of Bad Ischl Rosmoor, *E.V. Watson*, RNG (*M; m*); **4.** Tirol, Kufstein SE Kaiser. Hintersteiner Tal Wegrans, *J. Höller,* M (*M; m*; MF804339*, MF804349*, MF804344*); **5.** Tirol, Bezirk Reutte, Allgäuter Alpen *F. Schuhwerk* *94/124*, M (*M; m*; MF804341*, MF804346*, MF804351*). **France** **6.** Savoie, *Wallace*, RNG (*M*; *m*); **7.** Savoie, *C. Pin*, RNG (*M*). **Italy** **8.** Bormio, Val Pettin, *D.W. Tormaz*, NEU (*M*); **9.** Bormio, sentien de la Pliniana, *D.W. Tormaz,* NEU (*M*); **10.** Bormio, sur la rive ganche du Frodolfo, *D.W. Tormaz*, NEU (*M*; *m*); **11.** Bormio, marécauges au defrus de la rive ganche du Frodolfo, *D.W. Tormaz*, NEU (*M*); **12.** Dolomiten, Plan de Gralba, *G. Langer*, M (*M; m*; MF804342*, MF804347*, MF804352*). **Germany** **13.** Bayern, *A. Hemp & D. Podlech*, M (*m*); **14.** Bayern, S. Bächental, Lärchkogel, *J. Höller*, M (*M*; *m*; MF804338*, MF804343*, MF804348*); **15.** Bayern, Karwendelgebirge, *W. Lippert 26592*, M (*M*; *m*; MF804340*, MF804345*, MF804350*); **16.** Bayern, München, *H. Förther 4817*, M (*M; m*); **17.** Bayern, Unterhalb Fereinsalm, H. Löffelmann, RNG (*M*; *m*×2); **18.** Bayern, Enzianhütte, *J.Höller*, M (*m*). **Switzerland** **19.** Kanton Uri, *A. Kneucker*, RNG, *C. flava* var. *alpina* isolectotypus (*M; m*); **20.** Canton d’Appenzell, nord du Hirschberg, *A. Charpin & P. Geissler*, RNG (*M*; *m*); **21.** Valais, *P. Vargas*, MA (JN634687, JN627732, JN627799); **22.** Emosson, *C. Oberson*, NEU (*M; m*); **23.** Canton de Neuchâtel, Vallée de la Brévine, *J.P. Brandt*, NEU (*M*); **24.** Lignières, NEU (*M*; *m*).

**Dwarf morphotype from Pyrenees-Cantabrian Range** — **Andorra** **1.** Puerto de Envalira, *S.Castroviejo & B.Valdés-Bermejo*, MA (*M*); **2.** Lagos de El Serat, *M.Luceño & P. Vargas*, MA (*M*); **3.** Noguera de Ruda, *J.M.Marín* et al., UPOS (*M*); **4.** Lagos de Pesons, *J.M.Marín* et al., UPOS (*M*; *m*; JN634700, JN627744, JN627811). **France** **5.** Refugio de Valaz, *S.Muñoz*, MA (*M*); **6.** Hautes Pyrénées, Le Plan-Piau Engaly, *C.Aedo* et al., ARAN (*M*); **7.** Hautes Pyrénées, Lac de Badet, *C.Aedo &. J. Pedro*, MA (*M*; *m*; JN634695, JN627745, JN627812). **Spain** **8.** Asturias, Cantabrian Range, Puerto de Somiedo, *P.Jiménez-Mejías* et al., UPOS (*M*; *m*; JN634648, JN627746, JN627813); **9.** Cantabria, Cantabrian Range, Brañavieja, *C.Aedo*, MA (*M*); **10.** Cantabria, Cantabrian Range, Picos de Europa, El Cable, *M.Luceño*, MA (*M*); **11.** Cantabria, Cantabrian Range, Picos de Europa, Fuente Dé, *M.Luceño*, MA (*M*); **12.** Cantabria, Cantabrian Range, Fuente Dé, Áliva, *J.M.Marín et al*, UPOS (*M*×2; *m*; JN634698, JN627747, JN627814); **13.** Girona, Pyrenees, Vall de Ribes, Núria, *M.Luceño* et al., UPOS (*M*; *m*; JN634701, JN627749, JN627816); **14.** Huesca, Pyrenees, Los Lecherines, *Amich* et al., MA (*M*, *m*); **15.** Huesca, Pyrenees, Panticosa, *M. Luceño*, MA (*M*); **16.** Huesca, Pyrenees, Candanchú, *F.Gómiz*, LEB (*M*); **17.** Huesca, Pyrenees, Circo de Soaso, *M. Luceño*, MA (*M*); **18.** Huesca, Pyrenees, Benasque, *V.J.Arán & M.J.Tohá,* JACA (*M*); **19.** Huesca, Pyrenees, Canfranc, *L.Villar* et al., JACA (*M*); **20.** Huesca, Sallent de Gállego, *P.Montserrat* et al., JACA (*M*); **21.** Huesca, Pyrenees, Benasque, refugio de Ángel Orús, *P.Jiménez-Mejías* et al., UPOS (*M*); **22.** Huesca, Pyrenees, Panticosa, Lagos, *P.Jiménez-Mejías* et al., UPOS, (*M*; *m*×2); **23.** Huesca, Pyrenees, Panticosa, balneario, *P.Jiménez-Mejías* et al., UPOS, (*M*; *m*; , JN634693, JN627751, JN627818); **24.** Huesca, Pyrenees, Ordesa, Góriz, *P.Jiménez-Mejías* et al., UPOS (*M*; *m*); **25.** Huesca, Pyrenees, Ordesa, Monte Perdido, *J.Fernández-Arroyo* et al., UPOS (JN634694, JN627752, JN627819); **26.** Huesca, Pyrenees, Formigal *M. Portillo.*, UPOS (*M*; *m*); **27.** Huesca, Pyrenees, Ibón de Anayet, *P.Jiménez-Mejías* et al., UPOS (*M*; *m*); **28.** Huesca, Pyrenees, Sahún, *J.A.Sesé & J.V. Ferrández*, JACA (*M*); **29.** Huesca, Pyrenees, Bielsa, *J.V.Ferrández*, JACA (*M*); **30.** Huesca, Pyrenees, Panticosa, *P.Montserrat & J.A. Sesé*, JACA (*M*); **31.** Huesca, Pyrenees, Refugio de la Estiba, *J.A.Sesé & R. Jiménez*, JACA (*M*); **32.** Huesca, Pyrenees, San Juan de Plan, *J.V.Ferrández & J.A. Sesé*, JACA (*M*); **33.** Huesca, Pyrenees, Fanlo, Monte Perdido, *D.Gómez*, JACA (*M*); **34.** Huesca, Pyrenees, Castejón de Sos, *Tornero*, JACA (*M*); **35.** León, Cantabrian Range, Puerto de San Glorio, *J.Andrés & R. Carbó*, LEB (*M*); **36.** León, Cantabrian Range, Burón, *J.Andrés*, LEB (*M*); **37.** León, Cantabrian Range, Puerto de San Glorio, *J.Andrés et al*, LEB (*M*); **38.** León, Cantabrian Range, Puerto de Somiedo, *M.Mayor* et al., MA (*M*); **39.** León, Cantabrian Range, Peña Santa de Castilla, *S.Martín-Bravo*, UPOS (*M*); **40.** León, Cantabrian Range, Maraña, *E.Puente* et al., LEB (*M*; *m*); **41.** León, Cantabrian Range, Puerto del Pontón, *J.Andrés*, LEB (*M*); **42.** León, Cantabrian Range, Campohermoso, *M.J.López-Pacheco*, LEB (*M*); **43.** León, Cantabrian Range, Puerto de Leitariegos, *E.Fuentes*, LEB (*M*); **44.** León, Cantabrian Range, Aralla, *C.Pérez-Morales*, LEB (*M*); **45.** León, Cantabrian Range, Valdelugueros, *J.Andrés*, LEB (*M*); **46.** León, Cantabrian Range, Burón, *J.Andrés*, LEB (*M*); **47.** León, Cantabrian Range, Puerto de Vergarada, *Blanca*, LEB (*M*); **48.** León, Cantabrian Range, Monte de Tejedo, *J.Andrés & R.Carbó*, LEB (*M*); **49.** León, Cantabrian Range, Polvoredo, *J.Andrés*, LEB (*M*); **50.** León, Cantabrian Range, Pico Huevo, *J.Andrés et al*, LEB (*M*); **51.** Lleida, Pyrenees, Val d’Aran, Tredós, *J.M.Marín* et al., UPOS (*M*; *m*); **52.** Lleida, Pyrenees, Val d’Aran, Tredós, *M.Luceño* et al., UPOS (*M*; *m*); **53.** Lleida, Pyrenees, Val d’Aran, Les Bordes, *A.Pallarés*, MA (*M*); **54.** Lleida, Pyrenees, Val d’Aran, Baños de Tredós, *J.M.Marín & M.Luceño*, UPOS (*M*; JN634649, JN627753, JN627820); **55.** Palencia, Cantabrian Range, Curavacas peak, *J.Andrés*, LEB (*M*); **56.** Palencia, Cantabrian Range, Cardaño de Arriba, *C.Aedo* et al., MA (*M*).

**Dwarf morphotype from Sierra Nevada** — **Spain** **1.** Almería, Sierra de los Filabres, Gergal, *A.Pallarés*, MA (*M*; *m*); **2.** Almería, Sierra Nevada, Fiñana, *A.Pallarés*, MA (*M*; *m*); **3.** Almería, Sierra Nevada, El Chullo, *A.Pallarés*, MA (*M*; *m*); **4.** Almería, Sierra Nevada, Puerto de la Ragua, *A.Pallarés*, MA (*M*; *m*); **5.** Granada, Sierra Nevada, Corral del Veleta, *M.Luceño* et al., MA (*M*); **6.** Granada, Sierra Nevada, Monachil, *M.Luceño*, MA (*M*); **7.** Granada, Sierra Nevada, Hoya de la Mora, *J.Fernández-Casas*, MA (*M*); **8.** Granada, Sierra Nevada, Barranco del Goterón, *J.Fernández-Casas*, MA (*M*); **9.** Granada, Sierra Nevada, Prados de Otero, *M.Ladero* et al., MA (*M*); **10.** Granada, Sierra Nevada, Pico Veleta, *H.Merxmüller & W.Wiedmann,* M (*M*); **11.** Granada, Sierra Nevada, Pico Veleta, Barranco de San Juan, *S.Dürr*, M (*M*; *m*); **12.** Granada, Sierra Nevada, Laguna de las Aguas Verdes, *P.Jiménez-Mejías & M.Escudero*, UPOS (*M*; *m*); **13.** Granada, Sierra Nevada, Laguna de la Caldera, *M.Luceño* et al., UPOS (*M*×2*; m*); **14.** Granada, Sierra Nevada, Laguna de la Caldera, *M.Luceño* et al., UPOS (*M*; *m*×3); **15.** Granada, Sierra Nevada, Siete Lagunas, *P.Jiménez-Mejías* et al., UPOS (*M*; JN634647, JN627734, JN627801); **16.** Granada, Sierra Nevada, Laguna de la Mosca, *P.Jimenez-Mejías* et al., UPOS (*M*; , JN634646, JN627733, JN627800).

**Dwarf morphotype from the Atlas** — **Morocco** **1.** Jbel Angour, *S.L.Jury* et al., RNG (*M*); **2.** Oukaïmedem, *S.L.Jury* et al., RNG (*M*; *m*); **3.** Adrar-n-Oukaïmedem, *A.Herrero* et al., MA (*M*; *m*; JN634697, JN627736, JN627803); **4.** Marrakech, Oukaimeden, *D.Podlech*, MSB (*M*); **5.** Jbel Angour, *Davis*, MSB (*M*); **6.** Jbel Toubkal, Réfugé Nalbus, *W.Rauh*, M (*M*; *m*).

**C. demissa Hornem. subsp. demissa** — **Austria 1.** Wien, Bezirk, *B.Wallnöfer*, MA (JN634653, JN627690, JN627757;). **C. demissa subsp. cedercreutzii (Fagerstr.) Jac. Koopman** — **Azores 1.** São Miguel, *A. Hansen*, RNG (JN634677, JN627735, JN627802) **C. flava L.** — **Belgium 1.** Luxembourg, Bellefontaine, *M.Leten*, RNG (JN634682, JN627705, JN627772); **Bulgaria 2.** Rhodopes, *C. Navarro* et al., MA (JN634683, JN627706, JN627773); **Norway 3.** Troms, Hamneidet, *M. Luceño*, UPOS JN634689, JN627710, JN627777. **C. hostiana D.C.**— **Germany 1.** Bayern, Kreis Weilheim, *W.Lippert*, M (JN634715, JN627712, JN627779). **Spain 2.** Navarra, Baztan*, A.Balda* (JN634716, JN627714, JN627781). **C. lepidocarpa Tausch. subsp. lepidocarpa** — **Spain 1.** Valladolid, Encinas de Esgueva, *J.L.Fernández* MA (JN634706, JN627720, JN627787); **2.** Jaén, Cazorla, Hoya del Cambrón, *P.Jiménez-Mejías* et al., UPOS (JN634705, JN627719, JN627786). **Slovenia 3.** Nr. L. Bled, *H.J.M. Bowen*, RNG (JN634707, JN627716, JN627783). **C. lepidocarpa subsp. jemtlandica Palmgr.** — **Norway 1.** Hedmark, Furuberget, *K.Lye*, O (JN634710, JN627723, JN627790; **2.** Norway, Hedmark, Furuberget, *K. Lye*, O (JN634711, JN627724, JN627791). **C. viridula Michx.** — **Portugal** **1.** Coimbra, Lagoa das Braças, *M.Luceño*, MA (JN634670, JN627728, JN627795).
