# Supplementary figures and images for: Cut from the same cloth: The convergent evolution of dwarf morphotypes of the Carex flava group (Cyperaceae) in Circum-Mediterranean mountains
Source: PLoS One. 2017 Dec 27;12(12):e0189769. doi: 10.1371/journal.pone.0189769 (PMC5744957; doi:10.1371/journal.pone.0189769)

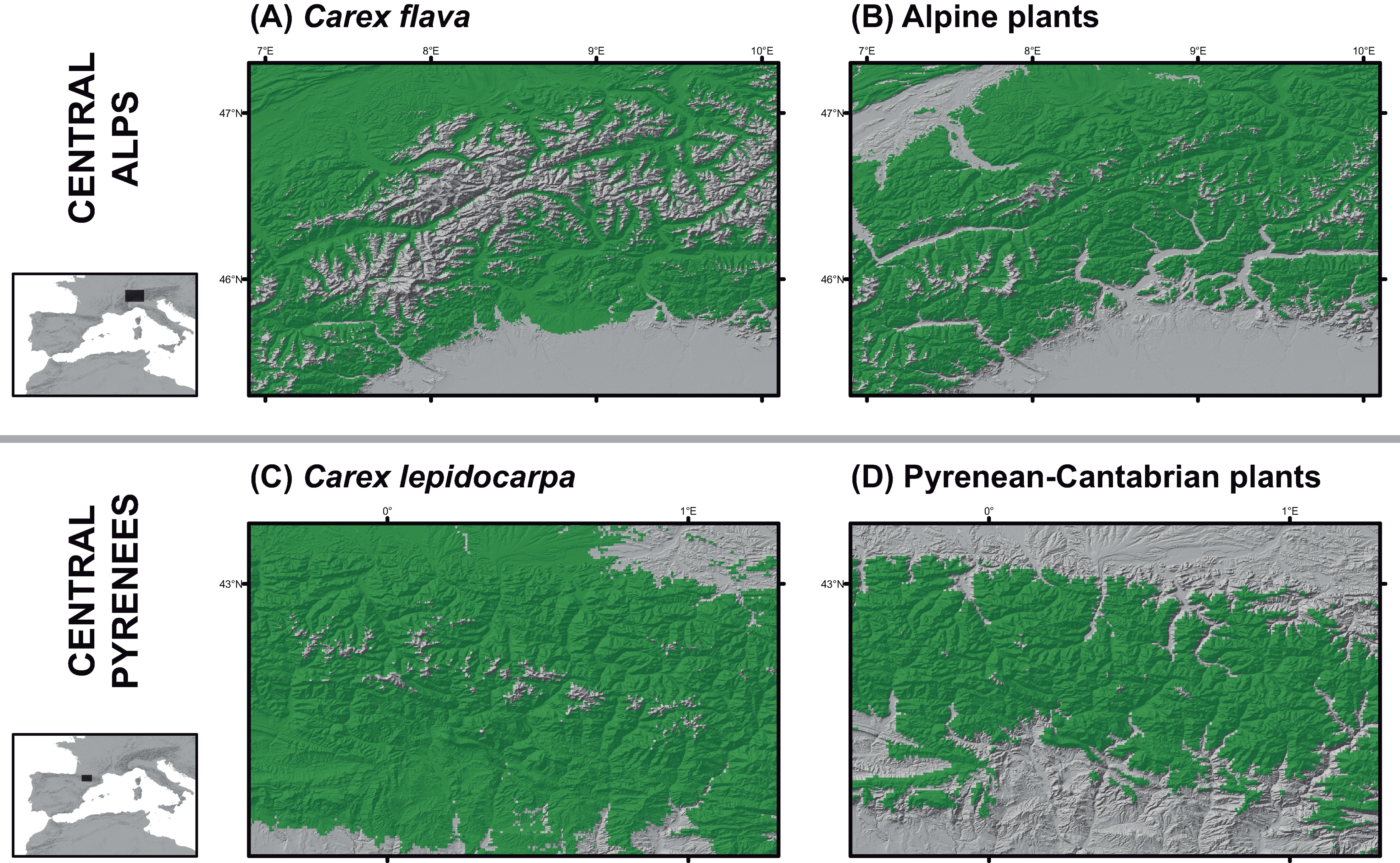

Supplement: S1 Fig — Details of distribution models of well-developed individuals and dwarf morphotypes in the central Alps (A, B) and the central Pyrenees (C, D). (TIF) [file pone.0189769.s003.tif]

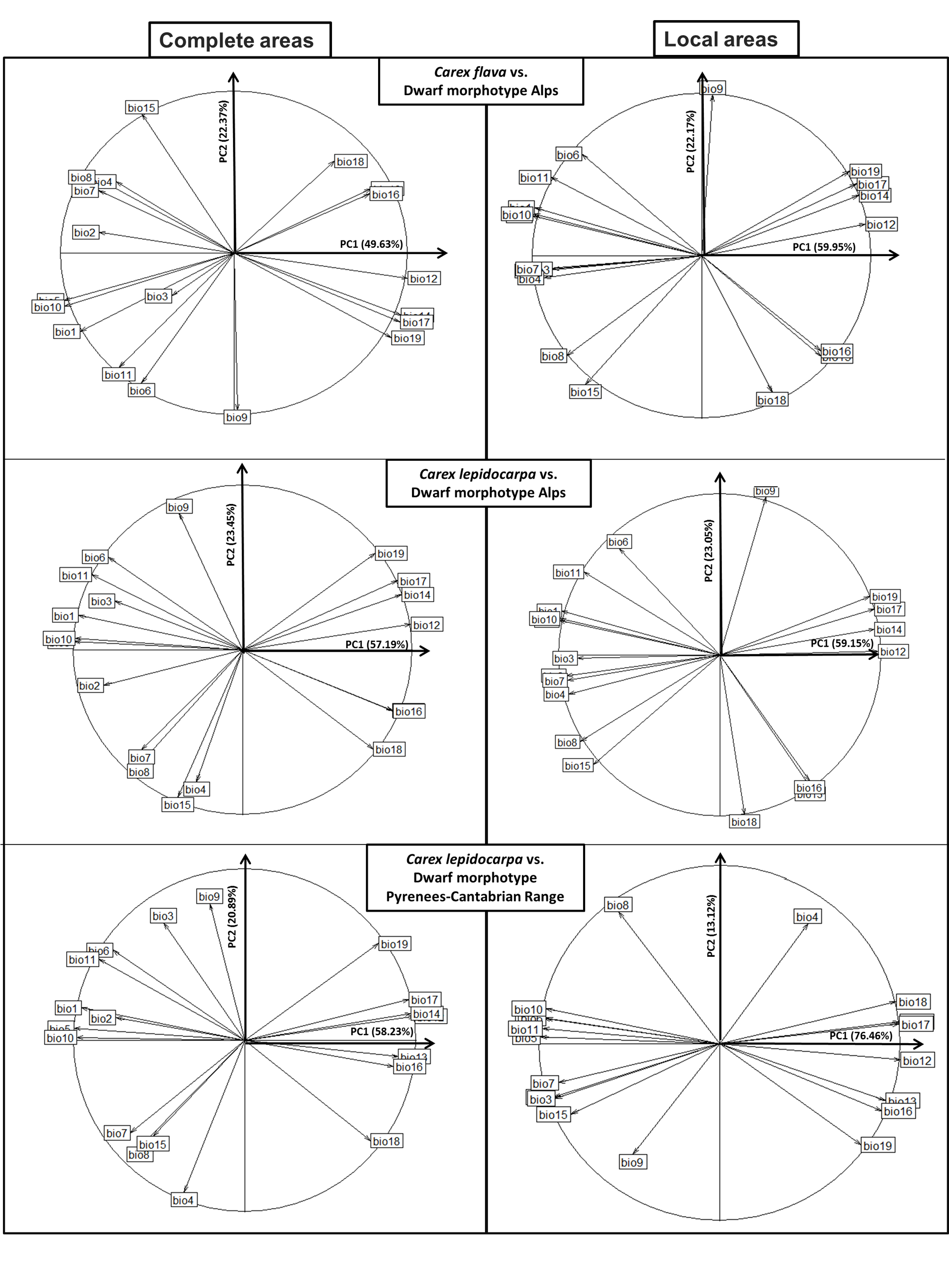

Supplement: S2 Fig — Relative contributions of the climatic variables to the two axes of the PCAs. in Fig 6. (TIF) [file pone.0189769.s004.tif]

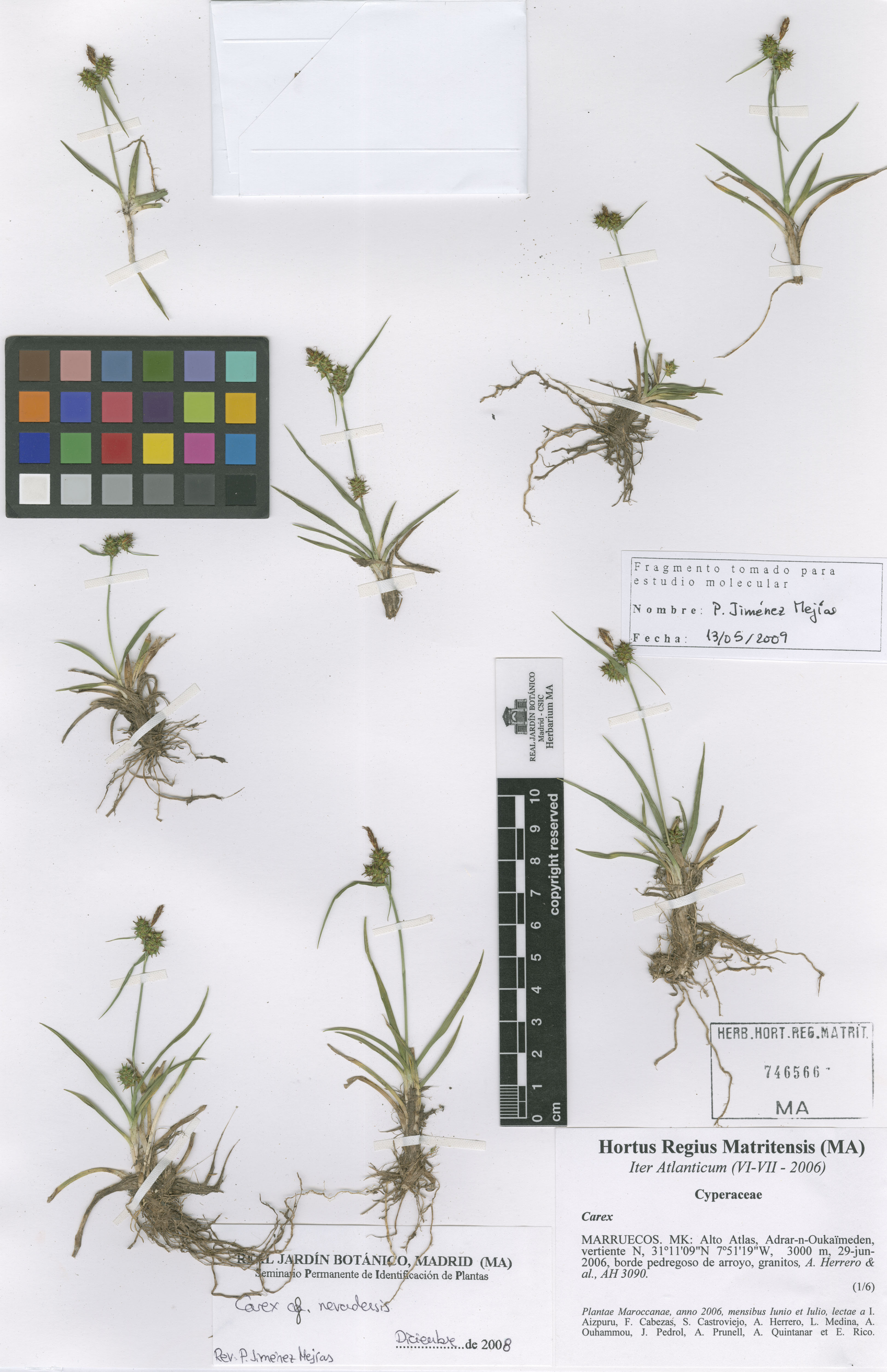

Supplement: S3 Fig — A. Herrero et al., AH3090, MA 746566. (TIF) [file pone.0189769.s005.tif]
