# Supplementary material for: Cut from the same cloth: The convergent evolution of dwarf morphotypes of the Carex flava group (Cyperaceae) in Circum-Mediterranean mountains
Source: PLoS One. 2017 Dec 27;12(12):e0189769. doi: 10.1371/journal.pone.0189769 (PMC5744957; doi:10.1371/journal.pone.0189769)
Supplement: S1 Table — Pairwise Pearson’s correlation coefficients between the 19 WorldClim bioclimatic variables calculated for a random sample of 1000 points of the study area (Europe and the Mediterranean region). (DOC) [file pone.0189769.s006.doc]

**Table S1.** Pairwise Pearson’s correlation coefficients between the 19 WorldClim bioclimatic variables calculated for a random sample of 1000 points of the study area (Europe and the Mediterranean region).

|  | bio01 | bio02 | bio03 | bio04 | bio05 | bio06 | bio07 | bio08 | bio09 | bio10 | bio11 | bio12 | bio13 | bio14 | bio15 | bio16 | bio17 | bio18 | bio19 |
| --- | --- | --- | --- | --- | --- | --- | --- | --- | --- | --- | --- | --- | --- | --- | --- | --- | --- | --- | --- |
| bio01 | 1.00 | 0.21 | -0.10 | 0.03 | -0.12 | -0.42 | -0.27 | -0.18 | 0.20 | -0.30 | -0.17 | 0.17 | 0.11 | 0.22 | -0.24 | -0.04 | -0.09 | -0.04 | -0.18 |
| bio02 | 0.21 | 1.00 | -0.66 | 0.19 | -0.64 | -0.45 | -0.11 | -0.07 | -0.36 | -0.23 | -0.43 | 0.22 | 0.31 | 0.33 | -0.44 | -0.24 | 0.21 | -0.17 | -0.05 |
| bio03 | -0.10 | -0.66 | 1.00 | -0.57 | 0.66 | 0.59 | -0.27 | 0.04 | 0.62 | 0.17 | 0.65 | -0.20 | -0.43 | -0.19 | 0.44 | 0.33 | -0.42 | 0.08 | 0.05 |
| bio04 | 0.03 | 0.19 | -0.57 | 1.00 | -0.08 | -0.36 | 0.66 | -0.15 | -0.46 | -0.04 | -0.50 | 0.10 | 0.34 | -0.15 | -0.12 | -0.26 | 0.32 | -0.07 | -0.15 |
| bio05 | -0.12 | -0.64 | 0.66 | -0.08 | 1.00 | 0.49 | 0.26 | -0.09 | 0.41 | 0.17 | 0.45 | -0.21 | -0.28 | -0.40 | 0.50 | 0.20 | -0.21 | 0.11 | -0.01 |
| bio06 | -0.42 | -0.45 | 0.59 | -0.36 | 0.49 | 1.00 | -0.11 | 0.10 | 0.24 | 0.18 | 0.49 | -0.16 | -0.28 | -0.33 | 0.43 | 0.24 | -0.20 | 0.06 | 0.09 |
| bio07 | -0.27 | -0.11 | -0.27 | 0.66 | 0.26 | -0.11 | 1.00 | -0.20 | -0.44 | 0.02 | -0.29 | -0.06 | 0.21 | -0.40 | 0.17 | -0.21 | 0.29 | -0.05 | 0.06 |
| bio08 | -0.18 | -0.07 | 0.04 | -0.15 | -0.09 | 0.10 | -0.20 | 1.00 | 0.00 | 0.19 | 0.23 | 0.01 | -0.09 | 0.19 | -0.04 | 0.02 | -0.02 | 0.09 | -0.02 |
| bio09 | 0.20 | -0.36 | **0.62** | -0.46 | 0.41 | 0.24 | -0.44 | 0.00 | 1.00 | 0.09 | 0.56 | -0.08 | -0.29 | 0.10 | 0.08 | 0.18 | -0.44 | 0.08 | -0.12 |
| bio10 | -0.30 | -0.23 | 0.17 | -0.04 | 0.17 | 0.18 | 0.02 | 0.19 | 0.09 | 1.00 | 0.07 | -0.14 | -0.21 | -0.11 | 0.14 | 0.11 | -0.13 | 0.05 | -0.01 |
| bio11 | -0.17 | -0.43 | 0.65 | -0.50 | 0.45 | 0.49 | -0.29 | 0.23 | 0.56 | 0.07 | 1.00 | -0.13 | -0.34 | 0.04 | 0.20 | 0.23 | -0.25 | 0.14 | 0.08 |
| bio12 | 0.17 | 0.22 | -0.20 | 0.10 | -0.21 | -0.16 | -0.06 | 0.01 | -0.08 | -0.14 | -0.13 | 1.00 | 0.28 | 0.03 | -0.16 | -0.28 | -0.02 | -0.04 | -0.22 |
| bio13 | 0.11 | 0.31 | -0.43 | 0.34 | -0.28 | -0.28 | 0.21 | -0.09 | -0.29 | -0.21 | -0.34 | 0.28 | 1.00 | -0.06 | -0.23 | -0.52 | 0.18 | -0.17 | -0.16 |
| bio14 | 0.22 | 0.33 | -0.19 | -0.15 | -0.40 | -0.33 | -0.40 | 0.19 | 0.10 | -0.11 | 0.04 | 0.03 | -0.06 | 1.00 | -0.54 | 0.04 | -0.13 | 0.09 | -0.11 |
| bio15 | -0.24 | -0.44 | 0.44 | -0.12 | 0.50 | 0.43 | 0.17 | -0.04 | 0.08 | 0.14 | 0.20 | -0.16 | -0.23 | -0.54 | 1.00 | 0.20 | -0.04 | 0.10 | 0.17 |
| bio16 | -0.04 | -0.24 | 0.33 | -0.26 | 0.20 | 0.24 | -0.21 | 0.02 | 0.18 | 0.11 | 0.23 | -0.28 | -0.52 | 0.04 | 0.20 | 1.00 | -0.19 | 0.15 | 0.31 |
| bio17 | -0.09 | 0.21 | -0.42 | 0.32 | -0.21 | -0.20 | 0.29 | -0.02 | -0.44 | -0.13 | -0.25 | -0.02 | 0.18 | -0.13 | -0.04 | -0.19 | 1.00 | 0.19 | 0.23 |
| bio18 | -0.04 | -0.17 | 0.08 | -0.07 | 0.11 | 0.06 | -0.05 | 0.09 | 0.08 | 0.05 | 0.14 | -0.04 | -0.17 | 0.09 | 0.10 | 0.15 | 0.19 | 1.00 | 0.02 |
| bio19 | -0.18 | -0.05 | 0.05 | -0.15 | -0.01 | 0.09 | 0.06 | -0.02 | -0.12 | -0.01 | 0.08 | -0.22 | -0.16 | -0.11 | 0.17 | 0.31 | 0.23 | 0.02 | 1.00 |
